# The test of basic Mechanics Conceptual Understanding (bMCU): using Rasch analysis to develop and evaluate an efficient multiple choice test on Newton’s mechanics

**DOI:** 10.1186/s40594-017-0080-5

**Published:** 2017-09-20

**Authors:** Sarah I. Hofer, Ralph Schumacher, Herbert Rubin

**Affiliations:** 1 School of Education, Technische Universität München, Arcisstr. 21, 80333 Munich, Germany; 2 MINT-Learning Center, ETH Zurich, Clausiusstrasse 59, CH-8092 Zurich, Switzerland

**Keywords:** Test construction, Newtonian mechanics, Rasch model, Conceptual understanding, Performance assessment

## Abstract

**Background:**

Valid assessment of the understanding of Newton’s mechanics is highly relevant to both physics classrooms and research. Several tests have been developed. What remains missing, however, is an efficient and fair test of conceptual understanding that is adapted to the content taught to secondary school students and that can be validly applied as a pre- and posttest to reflect change. In this paper, we describe the development and evaluation of the test of basic Mechanics Conceptual Understanding (bMCU), which was designed to meet these requirements.

**Results:**

In the context of test development, qualitative and quantitative methods, including Rasch analyses, were applied to more than 300 Swiss secondary school students. The final test’s conformity to the Rasch model was confirmed with a sample of *N* = 141 students. We further ascertained the bMCU test’s applicability as a change measure. Additionally, the criterion validity of the bMCU test was investigated in a sample of secondary school students (*N* = 66) and a sample of mechanical engineering students (*N* = 21). In both samples, the bMCU test was a useful predictor of actual student performance.

**Conclusions:**

The bMCU test proved to enable fair, efficient, and simultaneously rigorous measurement of secondary school students’ conceptual understanding of Newton’s mechanics. This new instrument might fruitfully be used in both physics classrooms and educational research.

**Electronic supplementary material:**

The online version of this article (10.1186/s40594-017-0080-5) contains supplementary material, which is available to authorized users.

## Background

A substantial part of students leaves school without having developed a proper understanding of basic physics concepts (see, e.g., Beaton et al. 1996; Halloun and Hestenes, 1985; McDermott, 1984). Science and particularly physics literacy, however, is becoming increasingly relevant in an environment that is based on scientific and technological progress. Groundbreaking work has been done by Halloun and Hestenes (1985) and Hestenes, Wells, and Swackhamer (1992), who were the first to systematically investigate learners’ naïve conceptions of mechanics, developed the well-known Force Concept Inventory (FCI; Hestenes et al. 1992), and thereby advanced the idea of learning as conceptual change. According to this line of research, when students enter the physics classroom for the first time, they have already built naïve conceptions of scientific phenomena to explain everyday experiences. These conceptions often are inconsistent with the scientifically accepted models that are taught at school; thus, these previously constructed conceptions can hamper learning (e.g., Duit, 2004; Hardy et al. 2006; Hestenes et al. 1992; Vosniadou, 1994). Familiar terms from everyday language that mean something completely different when used in the scientific context further lead to confusion—consider, for example, “force” and “work” (Brookes and Etkina, 2009; Rincke, 2011). With its many overlaps with everyday life, understanding Newtonian mechanics has proven particularly challenging (e.g., Halloun and Hestenes, 1985; Nieminen et al. 2010; Shtulman and Valcarcel, 2012). At the same time, however, basic Newtonian mechanics, which addresses how a body moves in response to forces acting upon it, provides the basis for later physics content.

To be able to ensure the acquisition of conceptual knowledge in basic Newtonian mechanics and to react to the students’ actual knowledge state and intervene appropriately, adequately assessing students’ understanding of Newtonian mechanics is highly relevant. Accordingly, in the following sections, we first introduce the general idea of conceptual knowledge that is closely linked to the best-known test of conceptual knowledge in physics, the FCI, which is addressed thereafter. We then show why there is need for a new instrument, ultimately setting the stage for the introduction of the test of basic Mechanics Conceptual Understanding (bMCU).

### Conceptual knowledge

Conceptual knowledge can be described as abstract and general knowledge of a domain’s main concepts and their connections (Carey, 2000; Schneider and Stern, 2010a). There is no unified definition of the single concepts that constitute conceptual knowledge (e.g., von Aufschnaiter and Rogge, 2015; Vosniadou, 2008). Researchers vary in the granularity that they apply when they speak about concepts. In the domain of physics, for example, “momentum conservation” or “force as deflector” have been considered concepts (see DiSessa, 1993; Halloun and Hestenes, 1985). In basic Newtonian mechanics, several interrelated concepts (e.g., “actio = reactio” and “inertia”) can be expected to form a higher level structure of conceptual knowledge that refers to the broad understanding of how a body moves in response to forces acting upon it. Following instruction in Newtonian mechanics, this knowledge structure is more or less elaborated, and the degree of understanding across all concepts reflects the state of a student’s conceptual knowledge of basic Newtonian mechanics.

Because prior knowledge determines the processing of new information (e.g., Carmichael and Hayes, 2001; Ohst et al. 2014; Stern, 2009), understanding new concepts depends upon the compatibility between the concept to be learned and existing conceptual knowledge. To enable learning in the event of incompatibility, conceptual change must occur (e.g., Posner et al. 1982; M. Schneider et al. 2012). Importantly, students’ prior conceptual knowledge that might not comply with scientifically accepted concepts must be considered important and productive in the course of the learning process. Conceptual change can encompass not only the replacement of certain elements but also the refinement and elaboration of existing knowledge structures (see Smith III et al. 1994; Vosniadou, 2008). Information about learners’ knowledge state is thus essential for effective instruction to explicitly work with and on students’ existing conceptual knowledge (c.f. M. Schneider and Stern, 2010a). Information about learners’ conceptual knowledge is important to judge whether they have truly understood the content taught. Because of its abstract nature, once acquired, conceptual knowledge enables flexible problem-solving that is not bound to specific contexts (see Hiebert, 1986). This type of deep understanding, more than problem-bound calculation routines or memorizing formulae, can be considered the essential element of physics literacy.

### Seminal role of the FCI

A major step in understanding students’ learning difficulties in physics was achieved when Hestenes, Wells, and Swackhamer (1992) presented their findings, which were gathered using the Force Concept Inventory (FCI; Hestenes et al. 1992; Halloun, Hake, Mosca, and Hestenes, 1995), a new type of assessment instrument. This test requires a choice between Newtonian concepts and naïve conceptions derived from everyday experience. Hestenes et al. (1992) demonstrated that even university students’ beliefs about the physical world are largely derived from personal experience and are often incompatible with Newtonian concepts. Since its publication, the FCI has been successfully applied in a large number of studies and has raised awareness of both the existence and persistence of naïve conceptions in diverse populations, including advanced physics students (e.g., Crouch and Mazur, 2001; Domelen and Heuvelen, 2002; Hake, 1998; Savinainen and Scott, 2002). To date, investigations of conceptual knowledge in a broad range of learning domains have been conducted (e.g., Hardy et al. 2006; Vosniadou, 1994), and further tests targeting heat and energy (Prince, Vigeant, and Nottis, 2012) or conceptual knowledge in biology (Klymkowsky and Garvin-Doxas, 2008) have been developed.

### Added value of a new instrument

Without undermining the FCI’s seminal contribution to educational research, there are conditions under which the FCI, along with a second well-known multiple-choice test in mechanics, the Force and Motion Conceptual Evaluation (FMCE; Thornton and Sokoloff, 1998), may be less appropriate for gauging understanding and learning. In the following sections, we refer to four criteria to demonstrate the need for an additional instrument that is adequate in situations in which the use of existing tests may be less appropriate.

#### Suitability for secondary school students

Because physics instruction in secondary school should ascertain the acquisition of basic physics literacy, it is important to have good measurement instruments that are specifically tailored to this group of learners in order to adequately react to students’ actual knowledge state and intervene appropriately. Both the FCI and the FMCE were designed to measure the conceptual understanding of a diverse population, from high school students to advanced university students. Existing research, however, suggests that these tests assess performance differences between younger learners less precisely than they do in a more advanced population. The Rasch model (that is introduced in more detail in the “Methods” section; Rasch, 1960) provides both a psychometric theory which can be used to guide the development of an instrument and techniques which can be used to investigate the measurement quality of the instrument. Planinic and her colleagues (2010) applied the Rasch model to FCI data on 1676 Croatian high school students with an average age of 17.5 years. They conclude “[…] that all items work together, but several problems are noticeable: poor targeting of the test on the population, too small separation of items in the middle of the test, too small width of the test, and the lack of easy items” (2010, p. 7). Data on German Gymnasium^1^ students from 13 classrooms who all worked on the FCI as pre- and posttest showed that, even at the posttest, Gymnasium students, on average, solved only 41% of the items correctly (Wilhelm, 2005). The FMCE’s suitability for secondary school students has not yet been specifically investigated. However, according to Thornton, Kuhl, Cummings, and Marx (2009), who compared the FCI and the FMCE, students’ percentage scores on the FCI tend to be higher than those on the FMCE suggesting that the latter could be the more difficult test (see also Redish, 2003, p. 102). Because both the FCI and the FMCE seem to be fairly difficult for secondary school students, they are not perfectly suited to precisely reflect individual performance differences at this school level.

To ascertain *content validity at secondary school level*, we involved secondary school teaching experts in the process of test development, avoided complex problem contexts, and explicitly adjusted the content to the subject material taught in the higher tracks of secondary school, which is where physics is more extensively instructed for the first time.

#### Efficiency

From a practical perspective that considers physics lessons lasting 40–45 min, crammed curricula, busy teachers, comprehensive test batteries in the context of educational studies, and respondent fatigue, efficient testing can be highly relevant. A short instrument that provides one index that validly measures conceptual understanding enables quick and exact testing, which is beneficial for both test takers who do not get tired and annoyed and for teachers or researchers who do not have to spend an excessive amount of time on the assessment, the analysis, and the interpretation of the test.

We developed a test that contains 12 items. The FCI, by contrast, consists of 30 items, which implies a longer working time and a higher level of mental effort both for those working on the test and for the person who must check the correctness and calculate the sum score of the 30 items. In addition, the FCI has been criticized as not truly measuring either a force concept or the six conceptual dimensions (including kinematics, the first law, or the superposition principle) that supposedly constitute the force concept, as indicated by factor analyses (Henderson, 2002; Huffman and Heller, 1995; Saul, 1998; Wilhelm, 2005; for a response of the FCI authors, see Hestenes and Halloun, 1995). Therefore, because it is not entirely clear whether the FCI sum score truly reflects one underlying construct, the sum score may not be a valid measure. The FCI enables a fine-grained analysis of students’ conceptual knowledge and naïve conceptions and thus unfolds its full potential when teachers or researchers have the resources and the intention to make use of this opportunity. However, if all they want is a single index that validly measures the students’ degree of conceptual understanding, applying the FCI does not really pay off.

With its 47 items, the FMCE is also not intended to provide an efficient, quick overview of students’ understanding but instead in-depth information about students’ conceptual knowledge and naïve conceptions in dynamics. It measures a conceptual understanding of Newton’s laws of motion with the following three factors (at least at the posttest; the factor analysis for the pretest is undefined): (1) “Newton’s first and second law, including acceleration,” (2) “Newton’s third law,” and (3) “velocity concept” (Ramlo, 2008). The instrument is thus best suited for analyses on either the factor level or even the single item level (see Thornton and Sokoloff, 1998).

To achieve *efficiency* (i.e., a quick and exact test), we aimed to choose a small number of items (quick) that conform to the Rasch model and therefore all measure the same underlying dimension (exact). In the Rasch model, simple sum scores can be used to easily determine a person’s ability level (see Boone and Scantlebury, 2006).

#### Suitability for measurement of change and fair measurement

Although both the FCI and the FMCE are routinely applied at several measurement points (often as pre- and posttest) to measure knowledge gain (e.g., Crouch and Mazur, 2001; Domelen and Heuvelen, 2002; Hake, 1998; Savinainen and Scott, 2002; Thornton and Sokoloff, 1998), recent analyses suggest that neither the FCI (Planinic et al. 2010) nor the FMCE (Ramlo, 2008) assess the same construct when applied as a pretest (without formal instruction on the topic or predominantly non-Newtonian sample, respectively) and a posttest (after instruction or predominantly Newtonian sample, respectively). According to Planinic et al. (2010), item parameters differed considerably between a predominantly non-Newtonian and a predominantly Newtonian sample, which should not have occurred if the same construct was measured in both samples. Ramlo (2008) reported that only at posttest did the FMCE factor structure closely resemble the factor structure found in earlier evaluations of the instrument. The factor structure at pretest was undefined, indicating that the FMCE measures two different constructs when applied as a pretest vs. a posttest. Accordingly, when FCI or FMCE pre- and posttest data are compared, change is assessed within uncertain frames of reference (see Cronbach and Furby, 1970; Lohman, 1999). We give one example to illustrate what we mean by uncertain frames of reference: the solution rate of an item might be very low because the meaning of certain language expressions is not yet known to the learners. Once the meaning has been clarified by the teacher, the solution rate might increase considerably. Change on this test might then reflect not only the test takers’ developing knowledge about the topic at hand but also their improved language skills.

Closely related to the previous point, all of the items of a test must measure the same construct for different groups of test takers to draw conclusions about inter-individual differences. When single items do not measure the same construct across subgroups (e.g., girls and boys), one speaks of differential item functioning (DIF). Using Rasch analyses, several FCI items were identified that function differently for males and females, for example (Dietz et al. 2012; Madsen, McKagan, and Sayre, 2013; Osborn Popp, Meltzer, and Megowan-Romanowicz, 2011). In addition to ensuring gender-fair measurement, there are also other groups of test takers (e.g., below-average and above-average intelligent students) that should be examined using item response theory (IRT) or the Rasch model to guarantee that an instrument enables fair inter-individual comparisons. This has not yet been done based on FCI or FMCE data.

We investigated whether our new test can provide *fair measurement* and constitutes a *valid change measure* by testing the fit of one uniform Rasch model both on data from different groups of test takers (e.g., female and male students) and on pre- and posttest data (see, e.g., Bond and Fox, 2007; Hambleton and Jones, 1993; Lord, 1980).

#### Filling a gap: the test of basic Mechanics Conceptual Understanding

To sum up, what is missing from the existing tests is an instrument whose content is adapted to the secondary school level in order to precisely assess performance differences between secondary school students (content validity at secondary school level). Because comprehensive instruments that diagnose specific concepts and naïve conceptions already exist, there is need for a user-friendly, quick, and exact test of conceptual understanding in mechanics (efficiency). We lack a test with confirmed suitability for inter-individual comparisons independent of the test taker’s individual characteristics (fair measurement), which not only enables one-time measurement but also can reflect learning progress (valid change measure). To satisfy these requirements, we drew on the Rasch model while constructing and evaluating a new instrument, the test of basic Mechanics Conceptual Understanding (bMCU). There is a growing number of studies on the evaluation of test instruments in the science education literature that are based on Rasch modeling (e.g., Cheng and Oon, 2016; Chiang, 2015; Kuo, Wu, Jen, and Hsu, 2015).

In addition, we inspected the bMCU test’s reliability interpreting item information curves and investigated its criterion validity. For the latter, we examined the bMCU test’s relationship to mechanics grades and to the FCI in a sample of secondary school students and a sample of mechanical engineering students. The bMCU test fills a gap and consequently complements and extends the scope of existing tests.

## Methods

**Fig. 1:**
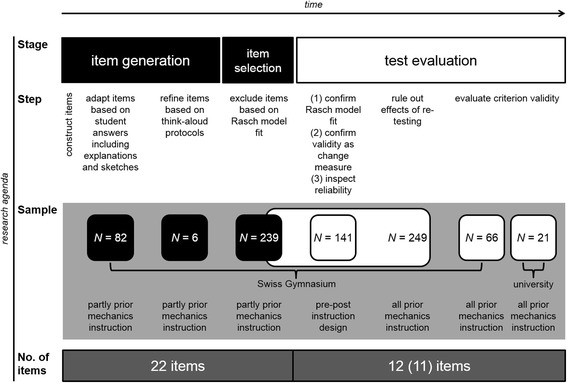
Schematic representation of the research agenda. Time flows from left to right. Corresponding information is vertically aligned. Rows provide information on the stage within the research process (first row), on the associated steps (second row), on the samples examined in each step (third, gray row), and on the number of items the respective sample worked on (fourth row)

Below, we first provide an overview of the Rasch model. The R packages applied and the instrument’s development, which consisted of two stages, item generation and item selection, are presented next. We then describe the strategy that we pursued to evaluate the final version of the bMCU test. Figure 1 gives an overview of the research agenda. For each of the three stages in the process of test development and evaluation (item generation, item selection, and test evaluation), the associated steps, the samples investigated, and the current number of items in the test are summarized. We provide a detailed description of the methods and analytic strategy to facilitate replication.

### The Rasch model

The dichotomous Rasch model^2^ is a psychometric model for binomial (dichotomous) data. The model assumes local stochastic independence and thus one-dimensionality, which means that all items of a test measure the same underlying construct. It further demands subgroup homogeneity, which means that all items of a test measure the same underlying construct across different subgroups (e.g., girls and boys). Moreover, the Rasch model claims that every item must contribute equally to the estimated ability level, implying equal item discrimination and thus, the absence of an item discrimination parameter. If all of these requirements are satisfied, a test instrument can be evaluated as unequivocally measuring a single underlying dimension (see, e.g., Bond and Fox, 2007; Strobl, 2010; Wright and Stone, 1979).

The Rasch model’s basic equation (see Eq. 1) describes the difference between the ability of a specific person n, *B*_*n*_, and the difficulty of a specific item *i*, *D*_*i*_, via a logarithmic function that depends upon the probability *P*_*ni*_ of person *n* to correctly solve item *i*:

1 $$ {B}_n-{D}_i=\ln \left({P}_{ni}/\left(1-{P}_{ni}\right)\right) $$

Thus, the person parameter (*B*_*n*_) represents a person’s ability level, and the item parameter (*D*_*i*_) represents an item’s difficulty. As indicated by the subtraction on the left side of Eq. 1, person and item parameters are measured on a single scale. A specific person’s probability *P*_*ni*_ of solving item *i* (right side of Eq. 1) is dependent upon the person’s ability *B*_*n*_ and the item’s difficulty *D*_*i*_ (left side of Eq.1). Consequently, if a specific person’s ability *B*_*n*_ complies with a specific item’s difficulty *D*_*i*_, the person’s probability of solving this item is *P*_*ni*_ = .50.

There are several methods available to test both the global fit of the Rasch model to the data and the fit between the data and the model’s assumptions of one-dimensionality, local stochastic independence, and subgroup homogeneity (see Strobl, 2010).

When a test fits the Rasch model, more able test takers on the trait that is measured are predicted to have a higher likelihood of correctly solving a specific test item than will lower-ability test takers attempting the same item. A person’s ability can thus be estimated by the number of the items of the test the person has solved correctly (i.e., the sum score). Consequently, a person’s sum score can legitimately be used to indicate a person’s ability level. This characteristic of a Rasch-scaled test instrument contributes to efficient testing because the test administrator can obtain a valid estimate of a person’s ability by simply counting the number of items solved correctly.

To obtain a conjoint measurement scale that applies to both person ability and item difficulty parameters, in the Rasch model, person and item parameters can be calculated by converting the raw scores into logits (logarithm of odds units; for more detailed information on parameter estimation, see, e.g., Linacre, 1998; Strobl, 2010).

### R packages

Throughout the test development and evaluation, R (R Core Team, 2013) was used to examine fit to the Rasch model. We applied the packages eRm (Mair, Hatzinger, and Maier, 2013) and ltm (Rizopoulos, 2006) for fitting and evaluating the Rasch model and generating item information curves. With the package nFactors (Raiche, 2011) and the R Stats Package, we confirmed one-dimensionality by means of factor analysis. We used the package WrightMap (Torres Irribarra and Freund, 2016) to draw Wright Maps and the package sirt (Robitzsch, 2014) to calculate Yen’s Q3.

### Item generation

The bMCU test was developed in a stepwise procedure with qualitative methods complementing quantitative item analyses. The following sections describe the construction of the items of the bMCU test, their adaption based on students’ answering patterns, explanations, sketches, and their refinement in reaction to think-aloud protocols of students’ reasoning (see Fig. 1). Whereas questions and answer alternatives were developed in the first step, the item adaption and item refinement steps were intended to optimally adapt the text of the questions and answer alternatives to the target population, to avoid ambiguities and misunderstandings, but not to select and exclude items. These two steps combined quantitative and qualitative analyses. The selection of the final number of items in the test (see section “Item selection”), however, should be based on a rigorous quantitative approach that assumes a strict measurement model (i.e., Rasch model analyses). This is why the number of items did not change during the item generation process (see Fig. 1).

#### Item construction

In the first step of test development, the focus was on arriving at a set of items with high content validity at secondary school level. Initially, a group of physics and secondary school teaching experts and educational psychology experts constructed a pool of 22 multiple-answer, multiple-choice items targeting introductory Newtonian mechanics. The physics and secondary school teaching experts provided a wealth of problem contexts and wrong answer alternatives inspired by their teaching experience. In addition, there is a great deal of research on naïve conceptions, particularly in Newtonian mechanics (e.g., Halloun and Hestenes, 1985; Hestenes et al. 1992; Thornton and Sokoloff, 1998). The 22 multiple-answer, multiple-choice items were constructed based on these resources. In this process, our group of physics experts, secondary school teaching experts, and educational psychologists discussed and estimated the difficulty of each item that was a function of the complexity of problem context and answer alternatives. We made sure that the items varied in difficulty.

Because the bMCU test should be particularly adapted to secondary school students, the problem contexts of the single items were less complex than were some of the problem contexts in the FCI, for instance. Problem contexts that require a great deal of information provided a priori were avoided. We aimed to construct items as concise as possible without hampering their comprehensibility. Because the bMCU test was also intended to serve flexibly as a pretest, it was important to avoid specific terminology that is difficult to understand without previous mechanics instruction (e.g., net force, normal force, and constant acceleration).

The items covered the topics “inertia and motion”, “force and acceleration”, “balance of forces”, and “reciprocal action”, which are routinely taught in introductory Newtonian mechanics at Swiss Gymnasiums that provide higher secondary education. These topics are also part of the recommendations and directives that define the secondary school physics curriculum in Switzerland (Gymnasium; e.g., Arbeitsgruppe HSGYM, 2008), Germany (Gymnasium; e.g., Bildungsstandards Physik Gymnasium, 2004; Lehrplan für das Gymnasium in Bayern-Physik 9, 2004; Lehrplan für das Gymnasium in Bayern-Physik 10, 2004), the UK (key stages 3 and 4; National curriculum in England-Science programmes of study: key stage 3, 2013; National curriculum in England-Science programmes of study: key stage 4, 2014), the US (high school; National Research Council, 2012), or Australia (high school; Physics senior secondary curriculum-unit 2: linear motion and waves, 2015; Science: Sequence of content, 2015). Therefore, Newtonian mechanics and the topics listed above are a regular feature of secondary school curricula.

To impede guessing, we built multiple-answer, multiple-choice items instead of single-answer, multiple-choice items. This approach enables us to survey deep conceptual understanding because students must detect all of the correct answers and omit all of the incorrect answers. Only then is an item scored *x*_*ni*_ = 1; otherwise, the item is scored *x*_*ni*_ = 0. If a student detects all of the correct answers and omits all of the incorrect answers of an item, the student can be expected to understand movement in response to forces in the particular problem context provided by the item. Only in this case, the item is scored 1. The selection of wrong answer alternatives and/or the omission of correct answer alternatives, in contrast, signify that a student does not yet possess the conceptual understanding necessary to solve the item correctly and the respective item is scored 0. Consequently, an item is either correct (1) or wrong (0). Due to the dichotomous categorization of bMCU test items as either correct or wrong, a person’s probability and complementary probability of solving each bMCU test item can be derived from counting correct items. Under the dichotomous Rasch model, the probability whether an item is solved correctly or not is determined by the ability of the person and the difficulty of the item (see Eq. 1). To estimate each person’s ability and each item’s difficulty, we can consequently apply the dichotomous Rasch model to the dichotomous bMCU test data.

**Fig. 2:**
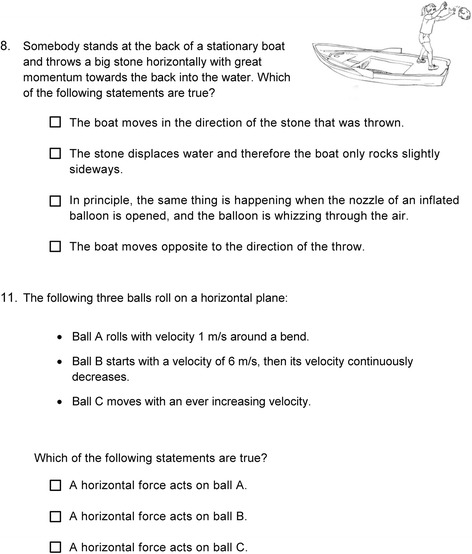
Two sample items of the Test of basic Mechanics Conceptual Understanding (bMCU) translated into English. For item 8 (“Stone”), the last two answer alternatives are correct, and for item 11 (“Balls”), all three answer alternatives are correct

For illustration, Fig. 2 shows two sample items that are included in the final version of the test. For item 8 (“Stone”), for instance, the last two answer alternatives are correct. Only if a student selects exactly these two answer alternatives, item 8 is scored 1. If the students select only the last answer alternative, for example, the item is scored 0.

The consistency with which a student can solve the items that address movement in response to forces across different problem contexts is assumed to reflect the student’s degree of conceptual understanding in Newtonian mechanics.

#### Item adaption

When the test was delivered to students, they always received the following general instruction: “In the following questions, more than one answer alternative may be correct. Check all correct answer alternatives. Please clearly mark your answers by checking the corresponding boxes. Please use a pencil and apply some pressure, ensuring that the cross is clearly recognizable. If you want to correct an answer, fully erase the cross in the box belonging to the wrong answer. Try to solve all questions. Do not dwell too long on a single question.” The instruction is also included in the test that is available as supplementary material accompanying the article (Additional file 1). In the first draft of the test, the 22 multiple-answer and multiple-choice items were supplemented by requests to explain the choice or to draw a sketch. The test was given to *N* = 82 Swiss Gymnasium students with and without instruction in mechanics. Students’ answering patterns, pictures, and comments were used to modify the items.

With regard to the answering patterns, we checked whether single answer alternatives were consistent with the averaged behavior of all answer alternatives. The solution rate of single answer alternatives was accordingly correlated with the averaged solution rates of all other answer alternatives. Answer alternatives with a low (or negative) correlation, which may indicate that an answer alternative is inconsistent with the other answer alternatives of the test (see, e.g., Everitt, 2002), were reformulated. Inconsistent answer alternatives were dropped only if there remained other answer alternatives that targeted the same content. If answer alternatives were chosen by almost all students or no student (i.e., very high or very low solution rate), they were critically revisited and dropped if they were considered too easy or too difficult for secondary school students. The students’ pictures and comments enabled conclusions about students’ naïve conceptions. Frequent naïve conceptions that had not yet been included in the test were added. The students’ drawings and explanations indicated that students sometimes misinterpreted the information given in the answer alternatives or questions. Critical text passages were reformulated to avoid ambiguities.

The analysis of the students’ comments and sketches in combination with their answering patterns further suggested that in some problem contexts, several students could correctly identify the correct result without having a correct understanding of the reason for that result. When it was not possible to reformulate or add answer alternatives to reflect these inconsistencies without creating highly complex answer alternatives, we decided to split the respective items into two parts (a and b). In the first part, students are thus asked what happens and in the second part, students are asked why it happens. Only if both parts are answered correctly can students be expected to have a correct conceptual understanding of Newtonian mechanics in the problem context at hand. Only in that situation is the item scored as one point.

The entire procedure was repeated several times until there were no remaining answer alternatives that were inconsistent, too easy, or too difficult, and no more pictures and comments that suggested insufficient intelligibility, ambiguities in the wording, or inconsistent measurement of conceptual understanding.

#### Item refinement

To finally ensure that our answer alternatives truly reflected students’ way of thinking, interviews were conducted with a sample of *N* = 6 (3 girls) Gymnasium students between 13 and 17 years with and without instruction in Newtonian mechanics. In the fashion of think-aloud protocols, the repeatedly modified set of 22 items was presented to the individual students without offering any answer alternatives (i.e., as open-ended items). Their answers and considerations were recorded and checked against the answer alternatives that we had constructed. We aimed to verify that all items could unambiguously differentiate between faulty thoughts and conceptual understanding. If the answers of at least one student indicated that an item could not clearly differentiate between faulty thoughts and conceptual understanding, we refined the respective item to unambiguously capture correct understanding.

The students’ thoughts reflected the answer alternatives constructed and suggested only minor further modifications. For instance, when examining item 8 (“Stone”), which is presented in Fig. 2, a student without prior instruction in mechanics and two students with prior instruction suggested that the water displaced by the stone moves the boat in the direction opposite to the direction the stone was thrown. They had a correct intuition for what is going to occur (boat moves in the opposite direction), but an incorrect explanation (waves). To be able to detect these faulty thoughts, we included a second correct answer alternative (“In principle, the same thing is happening when the nozzle of an inflated balloon is opened, and the balloon is whizzing through the air.”) to assess deep understanding of the underlying abstract principle. One student expected that nothing is going to occur, and two students expressed the correct idea. The students’ thoughts about the presented problem situation could hence be well mapped by the answer alternatives we had constructed, completed by the balloon analogy. Thus, we analyzed each item and the students’ thoughts about it.

Our group of physics experts, secondary school teaching experts, and educational psychologists again discussed and estimated the difficulty of each item to ascertain that the resulting set of items still covered different difficulty levels.

### Item selection

Having achieved a fixed set of good items based on the analyses applied in the first stage, in the second stage of test development, the aim was to ensure a fair, exact, and quick measurement of conceptual understanding of Newtonian mechanics (efficiency and fair measurement). Consequently, we checked the items for compliance with the Rasch model and excluded divergent items. Items could violate the Rasch model because they unintentionally assessed characteristics or abilities in addition to conceptual understanding of Newtonian mechanics or because of remaining ambiguities in the meaning of phrases or words, for instance. Testing for Rasch model fit enabled a quantitative examination of qualities of the items such as subgroup homogeneity, which could not be examined by the analyses in the first stage of test development. When single items do not measure the same underlying dimension across subgroups (e.g., native speakers and non-native speakers or girls and boys), one speaks of differential item functioning (DIF). Accordingly, to ascertain subgroup homogeneity, DIF must be avoided.

For this last step, the 22 items were distributed to a sample of *N* = 239 (150 girls) Swiss Gymnasium students with an average age of *M* = 16.34 (*SD* = 1.40, *range* 14–20) years. Information on age, gender, mother tongue, potential areas of specialization at school, and prior instruction in Newton’s mechanics were gathered. To detect DIF, we started with global tests and thus inspected Pearson’s χ^2^-goodness-of-fit (bootstrap) test (which assesses general model fit) and Andersen’s conditional likelihood ratio test (Andersen, 1973) along with the analogous non-parametric T10-statistic (Ponocny, 2001)^3^ with different splitting criteria. Andersen’s conditional likelihood ratio test and the T10-statistic examine the hypothesis that the item parameter estimation does not vary among subgroups. As splitting criteria, we used the bMCU mean, the bMCU median, the age median, gender, mother tongue (native speaker and non-native speaker), specialization (science and non-science), and prior instruction (yes and no). With the splitting criterion *gender*, for instance, item parameter estimates were compared between boys and girls. We expected no significant differences, given that the Rasch model holds. The splitting criterion *bMCU mean*, for example, implied that students scoring above average and students scoring below average on the bMCU test were compared in terms of item parameters.

**Fig. 3:**
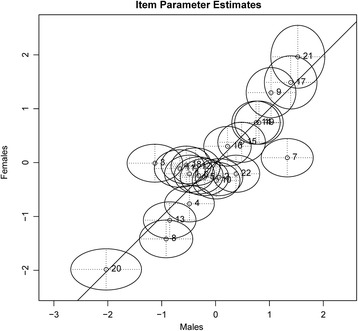
Exemplary graphical model test with 95% confidence regions and gender as splitting criterion. Item numbering does not correspond to the final item numbering. The estimated item parameters for males are plotted on the *x*-axis, and the estimated item parameters for females are plotted on the *y*-axis. Item parameter estimates are surrounded by 95% confidence regions. Significant deviation from the diagonal indicates an item’s subgroup heterogeneity. For items 3 and 7, parameter estimations differed markedly between males and females. Item 3, which addressed the deceleration of a car that is moving with constant speed, was easier to solve for males. Item 7, however, which addressed the interaction between the Earth and the Moon, was easier to solve for females

When at least one of the global tests indicated violations of the assumption of subgroup homogeneity, we continued with item-specific analyses (Additional file 2 “Additional Information on the Item Selection Process” that also includes a justification of our strategy to start with global and continue with local (i.e., item-specific) analyses is available as supplementary material). On the individual item level, the graphical model test with 95% confidence regions was conducted. This analysis estimates item difficulties separately for the two groups produced by the respective splitting criterion. The estimated item difficulties are plotted on two axes (i.e., each group on one axis) surrounded by confidence regions. An item’s subgroup heterogeneity is then indicated by significant deviation from the diagonal. Figure 3 provides an exemplary illustration of the graphical model test with the splitting criterion gender on the 22 items. It becomes clear that items 3 and 7 do not measure the same construct for males and females. Furthermore, the item-specific Wald test, which provides a significance test of the subgroup homogeneity assumption for each item, was inspected.

Whenever an item showed marked significant deviation or only slight discrepancies but on more than one statistic, the item was excluded. In a stepwise procedure, we eliminated the least fitting item first, repeated all of the tests and eliminated the next item. We stopped as soon as the tests indicated no further violations of the Rasch model. The resulting test consisted of 12 items with 3 to 10 answer alternatives each (item 4 “Train” and item 12 “Skaters” each comprise two parts, a and b). For students’ ease of processing, the final items were ordered by increasing difficulty. The ordering based on the item difficulty parameters was in line with the ordering based on the estimation of the items’ difficulties by our expert group, and the easy, moderate, and difficult items were included in the final version. The final version of the bMCU test, which still covers all of the topics “inertia and motion”, “force and acceleration”, “balance of forces”, and “reciprocal action”, is available as supplementary material (Additional file 1) accompanying the article.^4^

Thus, we developed a test of conceptual understanding of mechanics that is adapted to the content taught to secondary school students (content validity at secondary school level). By ascertaining Rasch model conformity, we identified a small number of items that nonetheless exactly measure understanding of Newton’s mechanics (efficiency) independent of several individual characteristics of the test taker (fair measurement).

The fit to the Rasch model (implying efficiency and fair measurement when homogeneity across different subgroups is ensured), however, had to be confirmed with a new sample of students who took the final 12-item version of the bMCU test. We also had to demonstrate the valid application of the bMCU test both as a pre- and a posttest to reflect learning progress (valid change measure) and examine the test’s reliability by interpreting item information curves. In addition, we had to rule out effects of re-testing that could influence the measurement. To finalize the instrument’s evaluation, the bMCU test’s criterion validity had to be ascertained. Consequently, we describe below our strategy to evaluate the bMCU test step by step (see Fig. 1).

### Test evaluation

#### Fit of the Rasch model, validity as a change measure, and reliability

In the following, we first present the student sample that was used to investigate the test’s fit to the Rasch model, its validity as a change measure, and its reliability. The steps taken to assess the final instrument’s fit to the Rasch model, substantiating its efficiency and its potential to provide fair measurement, and the strategy to examine the bMCU test’s validity as a change measure are delineated next. Finally, we briefly describe how we evaluated the bMCU test’s reliability.

##### Sample

The sample to confirm the fit of the bMCU test to the Rasch model, to investigate its applicability as a change measure, and to evaluate its reliability was taken from an ongoing research project. This research project implements cognitively activating Newtonian mechanics instruction (details follow in the next section) and compares this instruction with conventional instruction in real physics classrooms. All *N* = 141 (69 girls) participants with a mean age of *M* = 15.87 (*SD* = 1.10, *range* 14–19) years were Swiss Gymnasium students who took the bMCU test under supervision and without time pressure. In a maximum of 30 min, all of the students managed to work through the 12-item test. The bMCU test was administered before instruction (pretest) and after instruction in Newtonian mechanics (posttest). Unless otherwise specified, we always refer to the students’ bMCU posttest measure.

##### Assessing the fit of the Rasch model

Pearson’s χ^2^-goodness-of-fit (bootstrap) test assessed general model fit, whereas Andersen’s conditional likelihood ratio test and the nonparametric T10-statistic^5^ were applied to gauge subgroup homogeneity. We decided to examine DIF (i.e., homogeneity across different subgroups) using gender, type of instruction, and the medians of the bMCU measure, of age, and of intelligence as split variables.

#### Gender

It was considered especially important that the bMCU test measures boys and girls on the same scale. Gender-fair testing is essential in the context of performance assessment. Thus, we investigated DIF in terms of gender.

#### Type of instruction

A part of the sample (*n* = 58) received 18 lessons of introductory Newtonian mechanics instruction focusing on the conveyance of conceptual understanding. Methods such as metacognitive questions, self-explanations, holistic mental model confrontation, and inventing were implemented in this unit to help students grasp the meaning of the underlying concepts. We examined DIF between students who had received this type of cognitively activating instruction and students who had received conventional instruction (*n* = 83). We wanted to check whether different types of instruction differentially influence the probability of solving single items, which would ultimately change the meaning of the underlying construct that should be unambiguously measured independent of type of instruction. Generally, we ensured that there was no teaching to the test and that the content of the single items was not addressed during instruction.

#### bMCU measure

The median of the bMCU measure was used as a split variable to ascertain that the instrument does not function differently for students who have developed a relatively good understanding of the content compared to students with little or no such understanding.

#### Age

We also checked for DIF concerning age differences. Thus, younger students could be expected to solve items differently than older students, who might be further ahead in terms of their general cognitive development. The bMCU measure, however, should be directly related to conceptual understanding of mechanics, with any other influences ruled out.

#### Intelligence

Finally, a student’s intelligence level should not influence the items’ difficulty ranking and the test’s structure. Although intelligent students are expected to perform better than less intelligent students, differences in general intelligence should not lead to qualitative differences in how single items are solved. Intelligence was estimated using the set II score of Raven’s Advanced Progressive Matrices (Raven, Raven, and Court, 1992; maximum score = 36).

Another assumption of the Rasch model, one-dimensionality, was checked using the non-parametric version of the Martin-Löf test, which assesses whether different item-subsets all measure the same underlying dimension (see Verguts and De Boeck, 2000). This assumption could be violated, for instance, when effects of fatigue in the second half of the test systematically influence the measurement or when students learn from the first items. Thus, the first and second halves of the items were compared. The median of the item-specific solution rates was used as another criterion to split the items. In addition, odd items were compared with even items. A maximum-likelihood factor analysis with varimax rotation and the global non-parametric T11-statistic (Ponocny, 2001) that specifically tests for local stochastic independence were inspected to examine further whether a one-factor-solution fits the data and one-dimensionality can be warranted. Moreover, we calculated Yen’s Q3 (Yen, 1984) and followed the recommendations by Christensen, Makransky, and Horton (2017) for evaluating local stochastic independence.

Additional file 3 “Additional Information on the Test Evaluation Process” that provides further details on how Rasch model conformity was evaluated is available as supplementary material. Finally, item parameters (i.e., item difficulties) were estimated using the conditional maximum-likelihood method as implemented in the R package eRm (see Mair, Hatzinger, and Maier, 2013).

##### The bMCU test as a change measure

To examine whether the bMCU test measures the same latent dimension when applied as a pretest (without prior instruction) vs. posttest (with prior instruction in Newtonian mechanics), Andersen’s conditional likelihood ratio test and the non-parametric T10-statistic, which we had previously used as standard procedures to examine DIF, were inspected to compare the item parameter estimation between pretest and posttest data. Parameter estimation must not vary significantly as a function of time of measurement. When evaluating the bMCU test’s validity as a change measure, it was important to avoid dependencies in the data. Using random assignment, only the pretest data were analyzed for half of the participants, whereas only the posttest data were analyzed for the other half of the participants.

##### Reliability

In IRT and the Rasch model, measurement error can be computed for every level of person ability. By calculating a traditional reliability index, however, some information is lost because the possibly varying measurement error is averaged over different levels of person ability. Because the Rasch model enables consideration of the measurement error (or in other words, the precision of the measurement) at different levels of person ability, we present so-called item information curves that display the information provided by each item at each level of person ability. The interpretation of these curves is recommended over traditional approaches to evaluate reliability in IRT and Rasch models (see, e.g., Nicewander, 1993; Reise, Ainsworth, and Haviland, 2005; Robins, Fraley, and Krueger, 2009; Samajima, 1994; Thissen, 2000). Higher information corresponds to higher precision. For a dichotomous item in the Rasch model, the item information is *P*_*i*_ × (1 − *P*_*i*_), where *P*_*i*_ is the probability of correctly solving item *i*. Accordingly, the maximum value of each item information curve in the dichotomous Rasch model is 0.25 at *P*_*i*_ = .50. This means that an item’s information and thus its precision of measurement is highest for those persons whose ability complies with the item’s difficulty and who consequently have a probability of *P*_*i*_ = .50 of solving that item (see section “The Rasch model”). The inspection of the information curves of all items enables us to evaluate whether the test can provide precise measurement in the ability range of the target population or whether there are information gaps.

#### Effects of re-testing

In a pre-post instruction design, students complete the bMCU test repeatedly, as a pre- and as a posttest, which would be problematic if re-testing differentially influenced the probability of solving single items. To rule out effects of re-testing, we assessed DIF when one-time and repeated testing are compared.

##### Sample

To assess DIF when one-time and repeated testing are compared, we extended the sample of the *N* = 141 students by including parts of the sample of the *N* = 239 students whose data had been used in the stage of item selection in the test development process (see Fig. 1). Of the *N* = 239 students, only those with prior instruction in Newtonian mechanics were considered (*n* = 108). The sample to investigate effects of re-testing consequently consisted of *N* = 249 Swiss Gymnasium students with prior instruction in mechanics. Whereas the *n* = 108 students had worked on the bMCU test only once (i.e., one-time testing), the *n* = 141 students had already worked on the bMCU pretest (i.e., repeated testing).

##### Examining effects of re-testing

Again, Andersen’s conditional likelihood ratio test and the non-parametric T10-statistic were inspected to gauge DIF. If the item parameter estimation did not vary significantly between the two subsamples, it could be inferred that re-testing had no effect on Rasch model conformity. This information would guarantee that the test always measures the same underlying construct, whether applied as a posttest in a pre-post instruction design or for educational purposes only once at the end of a school year, for instance. However, note that the *n* = 108 students taken from the item selection sample had worked on the 22-item version of the instrument (see Fig. 1). Although we included the students’ scores on only the final 12 items, these 12 items could have measured a slightly different construct in the context of 10 additional items. Therefore, if item parameter estimation varied between the two subsamples, we could not infer whether this variation was caused by effects of re-testing or by effects of the use of different versions of the test instrument. Importantly, however, if item parameter estimation did not vary between the two subsamples, we could conclude that re-testing and the usage of the two versions of the test had no effect on the construct measured.

#### Criterion validity

To examine the criterion validity of the bMCU test, we investigated how the test predicted grades in Newtonian mechanics and how the test correlated with another concept test in the domain of Newtonian mechanics, the FCI. The successful prediction of grades, compared against the FCI, and a substantial correlation with the FCI can provide evidence that the bMCU test validly assesses students’ conceptual understanding of Newtonian mechanics.

##### Samples

For the evaluation of the bMCU test’s criterion validity, we used two samples, a sample of secondary school students and a sample of mechanical engineering students. Secondary school students were investigated because they are the target population of the bMCU test. University students were also considered to obtain a first idea of how the test functions in this more advanced sample. The secondary school student sample comprised *N* = 66 (38 girls) Swiss Gymnasium students from three physics classrooms with a mean age of *M* = 16.53 (*SD* = 0.66, *range* 15–18) years. The mechanical engineering students sample comprised *N* = 21 (2 girls) students in their first semester at the Swiss Federal Institute of Technology in Zurich. Both the secondary school students and the university students had recently addressed Newtonian mechanics in their classes.

##### Evaluating criterion validity

All of the participants worked on the final bMCU test and the German translation of the FCI by Gerdes and Schecker (1999) without time pressure. The order of the two tests was randomly interchanged so that half of the students in each school class and the university student sample initially worked on the bMCU test, whereas the other half worked on the FCI first. The number of items solved correctly (i.e., the sum score) for each test was used to predict the grade in Newton’s mechanics in the school student sample and the semester grade in Mechanics 1 (targeting Newton’s mechanics) in the university student sample. We also calculated the correlation between the two tests in both samples.

## Results

In the following sections, we present the findings of the evaluation of the bMCU test. First, the results concerning the fit of the Rasch model are provided. For readability, the finding concerning effects of re-testing on Rasch model conformity is included in this first section. The evaluation of the bMCU test as a change measure is outlined next. This chapter closes with the results of the reliability analysis and the assessment of the bMCU test’s criterion validity.

### Fit of the Rasch model

Concerning general model fit, Pearson’s χ^2^-goodness-of-fit (bootstrap) test suggested conformity of the data to the Rasch model (*p* = .19). Andersen’s conditional likelihood ratio tests and all of the nonparametric T10-statistics (including the examination of the effects of re-testing) indicated subgroup homogeneity, with all *p*s ≥ .07 (see Table 1). Consequently, there were no significant differences in the estimation of the item parameters between the subgroups that were compared (e.g., between girls and boys or between students who had worked on the test once and students who had worked on the test repeatedly). The test seemed to measure the same construct (i.e., conceptual knowledge in Newtonian mechanics) independent of the students’ gender, the type of instruction, the students’ performance on the bMCU test, the students’ age, the students’ intelligence, and one-time vs. repeated testing.

**Table 1:** Results of Andersen’s conditional likelihood ratio tests and the nonparametric T10-statistics with different split variables

Split variable	Subgroup size		*p* value Andersen	*p* value T10
	*n*_1_	*n*_2_		
Gender	69	72	.10	.08
Type of instruction	83	58	.23	.35
bMCU measure median	65	76	.07	.11
Age median	48	93	.71	.66
Intelligence (set II) median	61	64	.85	.67
Re-testing	108	141	.18	.18

*Notes*: Andersen’s conditional likelihood ratio test and the nonparametric T10-statistic gauge the homogeneity in the item difficulty parameter estimates between subgroups. The subgroups are determined by the six split variables. All non-parametric statistics are based on *n* = 5000 sampled matrices. A non-significant *p* value indicates no significant differences between subgroups in the item difficulty parameter estimation. *N* = 16 students of the total sample of *N* = 141 students were missing when intelligence was assessed. In the last row, the results of the examination of the effects of re-testing are presented. DIF is examined in a sample of *N* = 249 students when one-time (*n*_1_ = 108) vs. repeated testing (*n*_2_ = 141) are compared

The non-parametric version of the Martin-Löf test confirmed that all item-subsets tested against one another measured the same underlying dimension. Hence, the exact *p* value was estimated at *p* = .42 when comparing the first half of the items to the second half. Using the median of the item-specific solution rates as a split criterion, an exact *p* value of *p* = .13 resulted. The exact *p* value was *p* = .89 when comparing odd items to even items. In line with these results, a maximum-likelihood factor analysis with varimax rotation could substantiate the fit of the data of the bMCU test to a one-factor solution (χ^2^ = 59.63, *df* = 54, *p* = .28). The corresponding Scree plot (Additional file 4: Figure S1) and the factor loadings of the 12 items of the bMCU test given a one-factor, a two-factor, and a three-factor solution (Additional file 5: Table S1) are available as supplementary material accompanying the article. Moreover, the global non-parametric T11-statistic (*p* = .29) and Yen’s Q3 (maximum value of Q3 < .17) suggested model fit when testing for local stochastic independence. Consequently, how a student solved the items of the bMCU test depended solely upon the student’s ability. Other systematic influences on the student’s responses were ruled out and the assumption of one-dimensionality could be warranted. These findings concerning one-dimensionality further underpinned the validity of using only one index (i.e., one latent dimension) when conceptual understanding of basic Newtonian mechanics is assessed with the bMCU test.

The results concerning the item parameter estimation are presented in Table 2. To base the item parameter estimation on a sufficiently large dataset, we applied the sample used to examine the effects of re-testing (*N* = 249) because no influence of repeated vs. one-time testing on the estimation of the item parameters could be observed (see Table 1).

**Table 2:** Item difficulty *D*_*i*_, standard error of *D*_*i*_, 95% confidence interval of *D*_*i*_, and outfit mean-square (MNSQ) for the 12 items

Item	Item difficulty *D*_*i*_	Standard error	95% CI		Outfit MNSQ
			LL	UL	
1. Water glass	−2.12	0.17	− 2.46	− 1.79	0.92
2. Book	−1.24	0.14	− 1.52	− 0.97	0.97
3. Bus	−0.80	0.13	− 1.07	− 0.54	1.00
4. Train	−0.43	0.13	− 0.69	− 0.17	1.04
5. Hiker	−0.25	0.13	− 0.51	0.01	0.83
6. Cart	−0.04	0.13	− 0.30	0.22	1.12
7. Object motion	0.05	0.13	− 0.21	0.31	0.98
8. Stone	0.37	0.14	0.10	0.64	1.11
9. Inclined plane	0.58	0.14	0.30	0.85	0.96
10. Motorcycle	0.62	0.14	0.34	0.90	0.87
11. Balls	1.33	0.17	1.00	1.65	0.78
12. Skaters	1.95	0.20	1.57	2.34	0.84

*Notes*: *CI* confidence interval, *LL* lower limit, *UL* upper limit. Item difficulty parameter *D*_*i*_ and its standard error estimated according to the Rasch model. Higher positive values indicate higher difficulty. Confidence intervals provide an idea of the precision of the difficulty parameter estimation. Outfit MNSQ is a fit statistic comparing expected (based on the model) with observed data patterns that is sensitive to outliers. Values of approximately 1.00 (~ 0.50–1.50) indicate reasonable fit. The values obtained for the 12 items all fall within this range, indicating reasonable fit of the item data to the Rasch model (for information on less well-known conditional fit statistics, see Christensen and Kreiner, 2013; Müller and Kreiner, 2015)

**Fig. 4:**
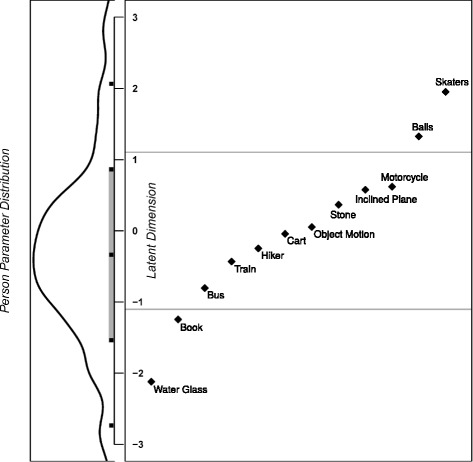
Wright Map on the 12 items of the bMCU test and the *N* = 249 students. The left panel provides the distribution of the students’ person parameters (ability), and the right panel depicts the location of each item’s difficulty parameter along the same latent dimension. From bottom to top, person ability and item difficulty increase. The gray bar on the latent dimension axis in the left panel indicates the range of values where approximately 68% of all person parameters are located. The black square in the middle of the bar marks the mean of all person parameters, the black squares at the upper and lower end of the bar correspond to the mean plus and minus one standard deviation. The two more extreme black squares on the latent dimension axis correspond to the mean plus and minus two standard deviations. Approximately 95% of all person parameters are located between these two squares. The two horizontal lines in the right panel correspond to the mean of the item parameters (i.e., *M* = 0) plus and minus one standard deviation, indicating the range of values where approximately 68% of all item parameters are located. The map shows that the items are reasonably spread across the students’ ability range, with most items covering the average ability range, in which most students are located

Figure 4 provides a Wright Map or person-item map (see, e.g., Bond and Fox, 2007; Boone, Staver, and Yale, 2014). This plot visualizes the location of each item’s difficulty parameter (right panel) together with the distribution of all person parameters (left panel) along the same latent dimension. The ordering of the items based on their locations on the latent dimension (i.e., their difficulty; see also Table 2) is in line with the ordering based on the estimation of the items’ difficulties by our expert group in the test development process. Comparing the item parameter distribution and the person parameter distribution, it becomes apparent that the items, which are reasonably spread across the latent dimension, cover large parts of the ability range of the students in the sample suggesting good test-item targeting. As indicated by the mean and standard deviation of the item difficulty (*M* = 0.00, *SD* = 1.10) and the mean and standard deviation of the person ability (*M* = − 0.34, *SD* = 1.20), with approximately 68% of all items being located between − 1.10 and 1.10 on the latent dimension and approximately 68% of the students’ ability parameters being located between − 1.54 and 0.86 on the latent dimension, the items concentrate on measuring the ability of a slightly more able student sample than the sample investigated. In the present student sample, however, the bMCU test was administered without relevant external incentives. The mean ability of average secondary school students could hence be expected to increase slightly when the test is applied in a situation that is more relevant to the students, resulting in an increased fit between item difficulty and person ability. Moreover, the bMCU test is also intended to be used for research purposes. Interventions may be designed to enhance the students’ performance. Therefore, good differentiation in average to higher ability ranges is important. Nevertheless, also in the present sample, the items are well suited to measure the ability of and differentiate between students in the average ability range, which is the range that is the most relevant. Simultaneously, a few items located at both the lower and upper ends of the latent dimension allow differentiation among especially low- and high-performing students. However, the more extreme regions of the latent dimension are less well covered.

### The bMCU test as a change measure

Andersen’s conditional likelihood ratio test and the nonparametric T10-statistic were inspected to compare the item parameter estimation under the Rasch model between pretest and posttest data. The *p* values resulting from both tests (all *p*s < .001) disproved the notion of one uniform (prior instruction-independent) Rasch model. The item-specific Wald test and the graphical model test, with 95% confidence regions, indicated that particularly for one item (item 2 “Book”), parameter estimations differed markedly (*p* < .001) between pretest and posttest. This item, which addresses normal force and its effect on a book lying on a table, was difficult for all of the students to solve correctly without instruction, but it was easy to solve correctly after instruction. In their everyday lives, students usually do not consciously recognize phenomena related to normal force. Without being introduced to this type of force in physics instruction, most students seem to have no idea about it. After this item was excluded, Andersen’s conditional likelihood ratio test and the non-parametric T10-statistic suggested conformity in the item parameter estimations between pretest and posttest (all *p*s ≥ .10). With the exception of item 2 (“Book”), the test therefore measures conceptual understanding of basic Newtonian mechanics on the same scale, or on the same latent dimension, for pretest and posttest data. Consequently, changes between pretest and posttest should be assessed with the 11-item version of the bMCU test. Following the previously described procedure, Rasch model conformity could also be ascertained for the 11-item version of the bMCU test. An additional “Results” section (Additional file 6 “Fit of the Rasch model for the 11-item version of the bMCU test”) and the corresponding Figures S2 and S3 (Additional files 7 and 8), along with the corresponding Tables S2, S3, and S4 (Additional files 9, 10, and 11), are available as supplementary material accompanying the article.

### The reliability and criterion validity of the bMCU test

**Fig. 5:**
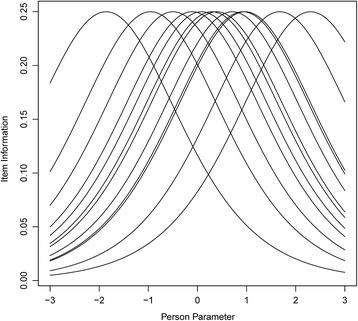
Item information curves for all items of the bMCU test as a function of the person parameters of the *N* = 249 students. Item information curves display the information provided by each item (*y*-axis) at each level of person ability (*x*-axis). Higher information corresponds to higher precision. The precision of the test at a certain level of person ability results from adding the information of each item at this level of person ability

Figure 5 presents the item information curves for all items of the bMCU test. For students with an ability (i.e., person parameter) between − 1 and 1 on the latent dimension, the bMCU test measures conceptual understanding with high precision. From − 2.5 to − 1 and 1 to 2.5, there are also no gaps in the information about person ability. In this range, however, the precision of the entire test is lower because fewer items provide information about person ability. The precision of the entire test at a certain level of person ability results from adding the information of each item at this level of person ability. For person parameters higher than 2.5 and in particular, lower than − 2.5, information decreases considerably. Consequently, in this range, person parameters are estimated less reliably and with a larger standard error of estimation. Referring to the Wright Map, however, we can conclude that the ability of most secondary school students is estimated reliably with approximately 68% of the students’ ability parameters being located between − 1.54 and 0.86 on the latent dimension and approximately 95% of the students’ ability parameters being located between − 2.74 and 2.06 on the latent dimension. The item information curves for the 11-item version of the bMCU test (see Additional file 12: Figure S4) closely resemble the item information curves for the 12-item version.

To examine the criterion validity of the bMCU test, we investigated how the test predicted grades in Newtonian mechanics, how this prediction compared against the prediction by the FCI, and how the bMCU test correlated with the FCI. We evaluated the bMCU test’s criterion validity in two samples. In the secondary school student sample, the bMCU measure and the FCI score correlated significantly with the grade in Newton’s mechanics (*r* = .48 and *r* = .38) and with one another (*r* = .63). The bMCU measure alone explained 23% of the variance in the grades (*p* < .001). When we added the FCI score, no significant change in the prediction was achieved. In comparison, the FCI score alone explained 14% of the variance in the grades (*p* < .01). When we included the bMCU measure in the regression, an additional 10% of the variance in grades could be explained (*p*_change_ < .01). In the regression with both predictors, only the bMCU measure significantly predicted grades.

In the mechanical engineering student sample, the bMCU measure but not the FCI score correlated significantly with the semester grade in Mechanics 1 (*r* = .56 and *r* = .26). The two tests again correlated significantly with one another (*r* = .67). The bMCU measure alone explained 32% of the variance in the grades (*p* < .05). When we added the FCI score, no significant change in the prediction was achieved. In comparison, the FCI score alone explained 7% of the variance in the grades (*p* = .26). When we included the bMCU measure in the regression, an additional 26% of the variance in the grades could be explained (*p*_change_ < .05). In the regression with both predictors, only the bMCU measure significantly predicted grades.

## Discussion

In this paper, we described the development and evaluation of a multiple-answer, multiple-choice test assessing fundamental conceptual understanding of Newton’s mechanics. The construction and evaluation of the instrument enabled us to create a fair, user-friendly, short, and exact test of conceptual knowledge (fair measurement and efficiency) that is adapted to the content taught to secondary school students (content validity at secondary school level) and that can be validly applied both as a pre- and posttest to reflect learning progress (valid change measure). The bMCU test can reliably estimate the ability of secondary school students in the average ability range in which most students are located. Because we aimed to provide an efficient test instrument with a small number of items, good coverage of the average ability range was considered more important than good coverage of the extreme regions.

We showed that the bMCU test significantly predicted mechanics grades not only in a sample of secondary school students but also in a sample of mechanical engineering students. Consequently, the bMCU test also proved to be a valuable predictor of university students’ understanding of mechanics, although it was not explicitly designed for this more advanced student group. Moreover, the bMCU test correlated significantly with another test of conceptual knowledge in Newtonian mechanics, the FCI. The strong correlation (*r* ~ .65) with this established test further underpinned the validity of the new instrument. In addition, the strong correlation between the bMCU test and the FCI was higher than the correlation between the bMCU test and grades. This finding suggests that differences in conceptual understanding, as measured by the two tests, are indeed reflected in grade differences but cannot fully explain the inter-individual variation in the grades (approximately 67% of the variance remains unexplained). This finding shows that the bMCU test has more in common with the FCI than with grades. While grades can be expected to capture additional components of achievement in physics (e.g., calculation skills), the bMCU test seems to focus on the students’ conceptual knowledge, similarly to the FCI. Moreover, the bMCU test proved a better predictor of grades than did the FCI in both samples, possibly indicating that the bMCU test is better adapted to the content taught to secondary school students. The mechanical engineering students were in their first semester at university. It seems that also the content covered in the introductory course they had attended (Mechanics I) is more closely related to the content covered by the bMCU test than to the content covered by the FCI. The finding that the bMCU test was a particularly useful predictor of student performance in both samples suggests a good coverage of introductory Newtonian mechanics that can be managed at the secondary school level. Interpreted together, these results offer sound arguments for the bMCU test’s validity. Below, we discuss the bMCU test’s implementation in educational practice and research before considering its limitations.

## Conclusions

### The bMCU test’s potential for physics instruction

The bMCU test can be easily implemented in the physics classroom. Test instructions are short and readily understandable by secondary school students. The test can be processed in approximately 20 min and analyzed in 1 min per test by simply checking answer alternatives and summing all of the items solved without mistakes (all of the correct answer alternatives and no wrong answer alternative marked). The distribution of the item parameters that represents each item’s difficulty enables a differentiated measurement of secondary school students’ abilities in the average achievement range with most items covering this area. Simultaneously, there are two easily solvable, encouraging items and two particularly difficult items that allow assessment at the top end and prevent ceiling effects. We ascertained that the instrument assesses the same underlying ability for girls and boys and for different age and intelligence groups. The test seems to measure conceptual understanding of Newton’s mechanics unambiguously, independent of both the quality of physics instruction and whether the test has been taken only once or repeatedly. Consequently, whenever fair and efficient assessment of understanding of Newton’s mechanics is required, the bMCU test may be considered. The test could not only complement summative assessments but also be highly valuable in the context of formative assessment (e.g., Centre for Educational Research and Innovation, 2005; Wiliam, 2010), in which efficiency is especially important.

### The bMCU test’s potential for research

Because the bMCU test provides efficient and fair one-time measurement as well as measurement of change in samples of secondary school students, it can be broadly applied in research on physics learning as outcome or predictor variable, for instance. The new instrument constitutes a valuable instrument for assessing the effects of interventions in the context of Newton’s mechanics. It can be used to compare different instructional approaches, guaranteeing a fair measurement without qualitative differences in item processing and conceivability. Fair measurement results from Rasch conformity and has been tested, for example, when comparing item parameter estimates for students who had received cognitively activating instruction vs. conventional instruction. One major advantage of the bMCU test compared to existing instruments is its confirmed applicability as a change measure. There is evidence that item 2 “Book” might be inappropriate when comparisons over time are intended. To measure change, this question should be excluded. The resulting 11-item version of the bMCU test, however, measures the same underlying construct independent of students’ prior instruction. Thus, the 11-item version can be validly applied as both a pre- and a posttest to reflect learning progress.

There is no need to eliminate item 2 in general. When the bMCU test is applied only once and no comparisons over time are intended (which is probably most often the case when the test is used in physics instruction), item 2 is valuable because it provides further information about the students’ conceptual understanding. In such cases, the 12-item version is applicable. Researchers and practitioners must decide which version to use depending on the area of application and the corresponding requirements on the instrument.

In this paper, we show that it is legitimate to consider conceptual understanding of basic Newtonian mechanics on one dimension, resulting in a single index. This characteristic of the bMCU test recommends the instrument for all types of research projects that require a single, efficient measure of students’ conceptual understanding of mechanics.^6^

Hofer, Schumacher, Rubin, and Stern (2017) describe a classroom intervention study where the bMCU test was used to assess secondary school students’ conceptual understanding in mechanics. The bMCU test was also applied to measure the prior conceptual knowledge in Newtonian mechanics of first-year students at the Swiss Federal Institute of Technology in Zurich (see the ongoing project EQUATES—“equal talent, equal success”).

### Limitations and outlook

Although our results seem quite stable, our sample sizes were only moderate. When item parameter estimates are to be used to define competence levels or thresholds for later use, for instance, item difficulties must be highly precise. With larger samples, the difficulties of all items can be estimated more precisely. Nevertheless, it has been shown that the Rasch model is applicable to small sample sizes. Even sample sizes of approximately 35 have been considered sufficient to calibrate an instrument based on the Rasch model (see Linacre, 1994; Lord, 1980; Wright and Stone, 1979).

Whereas the total sample sizes used to fit the Rasch model (*N* = 239) and confirm the fit of the Rasch model (*N* = 141) can accordingly be considered appropriate, the results concerning DIF might need further validation. The subgroup sample sizes have been reported as partially insufficient to guarantee reliable detection of DIF (see Koller, Maier, and Hatzinger, 2015; Scott et al. 2009). However, we also applied non-parametric statistics that facilitate reliable detection of DIF even in small samples (see Ponocny, 2001). We provide a first positive evaluation of the bMCU test, but we hope that future uses of the test will consolidate our findings.

Moreover, we primarily investigated secondary school students from Switzerland. Examining additional secondary school student populations from other countries is thus needed. In particular, it remains to be determined whether the psychometric properties of the English version of the bMCU test, applied to an English-speaking population, are comparable to the psychometric properties of the original German version, applied to a Swiss-German population. The bMCU test’s potential for application at the university level could also be investigated further with larger and more diverse samples.

We have already initiated follow-up data collection in high schools and college in the USA, in the UK, in Australia, and in Germany. The results of this large international comparison study will be reported in detail in a separate paper. After the first step of sharing this new instrument and communicating its development and evaluation, we (and others who consider the test useful, we hope) will apply it and advance our knowledge.

In conclusion, the bMCU test proved to enable fair, efficient, and simultaneously rigorous measurement of secondary school students’ conceptual understanding of Newton’s mechanics. This new instrument might fruitfully be used in both physics classrooms and educational research.

## Additional files

Additional file 1: English version and the original German version of the bMCU test. (ZIP 314 kb)

Additional file 2: Additional Information on the Item Selection Process. (PDF 349 kb)

Additional file 3: Additional Information on the Test Evaluation Process. (PDF 353 kb)

Additional file 4: Figure S1. Scree plot showing the eigenvalues for different factor solutions on bMCU test data. (PDF 38 kb)

Additional file 5: Table S1. Factor loadings of the 12 items of the bMCU test given a one-factor, a two-factor, and a three-factor solution based on the sample of *N* = 249 students. (PDF 55 kb)

Additional file 6: Fit of the Rasch model for the 11-item version of the bMCU test. (PDF 318 kb)

Additional file 7: Figure S2. Scree plot showing the eigenvalues for different factor solutions on the 11-item version of the bMCU test. (PDF 39 kb)

Additional file 8: Figure S3. Wright Map on the 11-item version of the bMCU test and the *N* = 249 students. (PDF 80 kb)

Additional file 9: Table S2. Results of Andersen’s conditional likelihood ratio tests and the nonparametric T10-statistics with different split variables for the 11 items of the 11-item version without item 2 “Book”. (PDF 231 kb)

Additional file 10: Table S3. Factor loadings of the 11 items of the 11-item version of the bMCU test given a one-factor, a two-factor, and a three-factor solution based on the sample of *N* = 249 students. (PDF 55 kb)

Additional file 11: Table S4. Item difficulty *D*_i_, standard error of *D*_i_, 95% confidence interval of *D*_i_, and outfit mean-square (MNSQ) for the 11 items of the 11-item version without item 2 “Book”. (PDF 168 kb)

Additional file 12: Figure S4. Item information curves for the 11 items of the 11-item version of the bMCU test as a function of the person parameters of the *N* = 249 students. (PDF 60 kb)

## Abbreviations

bMCU test: basic Mechanics Conceptual Understanding test
DIF: Differential item functioning
FCI: Force Concept Inventory
FMCE: Force and Motion Conceptual Evaluation
IRT: Item response theory

## Footnotes

1 In Germany and in Switzerland, the Gymnasium is a public school that provides higher secondary education to above-average achieving students. The Gymnasium constitutes the highest track of the German and Swiss educational system. Gymnasium students (approximately 20–25% of all Swiss students) are comparable to US high school students attending college preparatory classes.

2 When we discuss the Rasch model in the following, we always refer to the dichotomous Rasch model. However, there are also other applications of Rasch theory (e.g., the polytomous Rasch model).

3 Non-parametric tests, which make no assumptions about underlying probability distributions, provide results that are more robust than the usual parametric tests against violations of common assumptions of inferential statistical tests.

4 The test is provided in the original German version and in a translated English version. The English version was translated by professionals in both languages with a science background. In addition, an English native speaker with expertise in physics was consulted to discuss the translation.

5 All of the non-parametric statistics were based on *n* = 5000 sampled matrices.

6 If desired and if efficiency is no issue, the bMCU test can, of course, also be interpreted on the single answer alternative level. In that event, more specific diagnoses about students’ conceptions regarding the contents and problem contexts covered by the respective item are possible.
