# Supplementary material for: The test of basic Mechanics Conceptual Understanding (bMCU): using Rasch analysis to develop and evaluate an efficient multiple choice test on Newton’s mechanics
Source: Int J STEM Educ. 2017 Sep 20;4(1):18. doi: 10.1186/s40594-017-0080-5 (PMC6310380; doi:10.1186/s40594-017-0080-5)
Supplement: Supplementary file 1 — English version and the original German version of the bMCU test. (ZIP 314 kb) [file 40594_2017_80_MOESM1_ESM.zip › bMCU_Test_English.pdf]

Dear test administrator,

Item 2 “Book” might be inappropriate when comparisons over time are intended and the bMCU Test is applied repeatedly because it might not measure the same underlying construct for students with and without prior instruction. Consequently, **to measure change, item 2 should be excluded**, and the resulting 11-item version should be used.

When no direct comparisons over time are intended, the full 12-item version is applicable.

Important note:

In the following questions, **more than one answer alternative may be correct**. Check all correct answer alternatives.

Please clearly mark your answers by checking the corresponding boxes: ☒

Please use a pencil and apply some pressure, ensuring that the cross is clearly recognizable. If you want to correct an answer, fully erase the cross in the box belonging to the wrong answer.

Try to solve all questions. Do not dwell too long on a single question.

1. A glass full of water stably rests on the back seat of a car that moves straight ahead with constant velocity. Suddenly, the driver presses the gas pedal and the car accelerates. Which of the following statements are true?

   - ☐ Because the glass does not move with respect to the back seat of the car, the water surface does not change.
   - ☐ The water is accelerated with the car, and therefore some water spills over the rim in the direction of travel.
   - ☐ Due to inertia the water surface does not change.
   - ☐ The water initially retains its previous state of motion and therefore some water spills over the rim against the direction of travel.

2. A book lies in front of you on the table. Which of the following statements are true?

   - ☐ Like any other resting object it is only affected by the gravity of the earth.
   - ☐ The table supports the book and therefore affects it with an upwards directed force.
   - ☐ As the book is stationary the term force does not apply in this case.
   - ☐ Only the supporting force of the table is acting on the book as it would otherwise fall to the ground.

3. A bus drives at a constant speed straight ahead on a horizontal road. Which of the following statements are true?

   - ☐ For the bus not to slow down, the propulsive force has to be equal to the air resistance and all the other frictional forces together.
   - ☐ For the velocity to remain constant, the propulsive force has to be greater than the air resistance and all other frictional forces together.
   - ☐ In order for the velocity not to increase, the propulsive force has to be slightly less than the air resistance and all other frictional forces together.
   - ☐ Propulsive force is only required for acceleration, however not at constant velocity.

4. a) A boy plays with a ball in the aisle of a railway carriage of a train that travels with constant velocity. Which of the following statements are true?

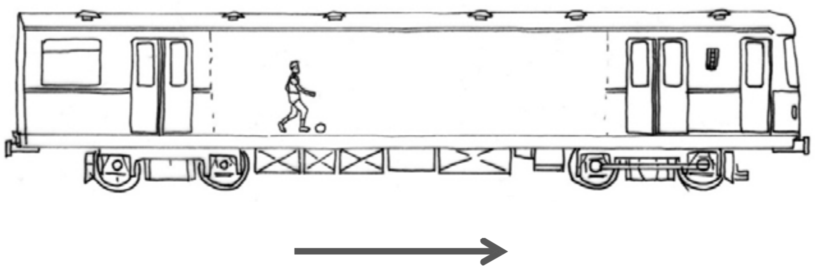

- ☐ Depending on whether he kicks the ball from the middle of the carriage in the direction of travel or against the direction of travel, the ball will arrive at the respective end of the carriage after different amounts of time.
- ☐ If he throws the ball straight up he needs to run either in the direction of travel or against the direction of travel to catch the ball again.
- ☐ The ball always behaves as if the train was stationary.
- ☐ Depending on whether he kicks the ball in the direction of travel or against the direction of travel he needs different amounts of force for the kick.

b) On which of the following explanation(s) have you based your answer(s)?

- ☐ As soon as the ball is thrown upwards its movement stays behind with respect to the movement of the carriage. The reason for this is that the inertia of the ball resists a change of movement.
- ☐ When the ball is thrown upwards the vector of the vertical movement is added to the vector of the horizontal movement in the direction of travel. Thus the ball moves away from the boy in the direction of travel.
- ☐ To kick the ball against the direction of travel one needs more force than when kicking it in the direction of travel. This is because when kicking against the direction of travel one has to kick, in addition, against the ball's direction of movement.
- ☐ Due to inertia the ball will always travel with the same horizontal speed as the carriage, except it is kicked towards or against the direction of travel.
- ☐ When the ball is kicked from the middle of the carriage towards the front, the front part of the carriage moves away from the ball. When the ball is kicked towards the back, the back end of the carriage is moving towards the ball. Therefore, the times taken by the ball to reach the respective ends of the carriage are different.

5. A hiker picks up a stone and continues walking at a speed of 1 m/s. After a short while he drops the stone again from a height of 1 meter while walking. After 0.5 seconds the stone hits the ground. Where does the stone hit the ground?

- ☐ The stone lands about 0.5 meters behind the hiker, because in 0.5 seconds the hiker walks approximately 0.5 meters.
- ☐ The stone lands approximately next to the hiker's feet, because the stone retains its horizontal movement due to inertia.
- ☐ Since the stone falls to the ground on a backward curved trajectory, it hits the ground approximately 1 meter behind the hiker.
- ☐ Since the stone retains its horizontal movement due to inertia, it hits the ground about 0.5 meters in front of the hiker.

6. On a model railway cart rests a metal ball that can roll unhindered to the left and right on a track on top of the cart. There are buffers at both ends of the track to prevent the ball from rolling off the cart.

Initially the ball is in the middle of the cart.

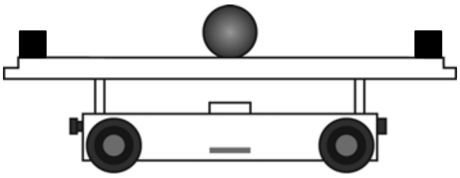

What happens if the cart is pushed from stationary towards the right?

- ☐ The ball rolls on the track to the left, against the direction of travel.
- ☐ The ball does not change its position on the cart.
- ☐ The ball rolls to the right in the direction of travel.
- ☐ The ball remains at approximately the same position with respect to the ground, until it hits the buffer on the cart.

7. After an object is pushed it slides on top of a smooth surface without any friction and air resistance. Which of the following statements are true?

- ☐ The momentum of the push is used up over time. Therefore the object becomes slower and slower until it comes to a halt.
- ☐ As the object is moving it is affected by a force aligned with the direction of movement.
- ☐ The mass of the object resists movement. The heavier the object the sooner it will come to a halt.
- ☐ The object glides with constant velocity across the surface.
- ☐ The object does not change its movement, because no horizontal force acts on it.

8. Somebody stands at the back of a stationary boat and throws a big stone horizontally with great momentum towards the back into the water. Which of the following statements are true?

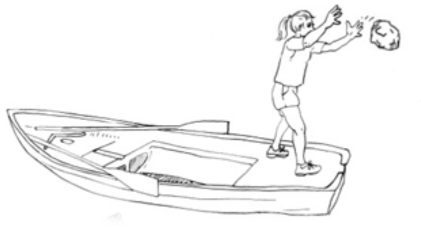

- ☐ The boat moves in the direction of the stone that was thrown.
- ☐ The stone displaces water and therefore the boat only rocks slightly sideways.
- ☐ In principle, the same thing is happening when the nozzle of an inflated balloon is opened, and the balloon is whizzing through the air.
- ☐ The boat moves opposite to the direction of the throw.

9. The following four figures show a ball that moves frictionless on different straight or curved trajectories. In which figures are the forces that act on the ball changing over time in the course of the movement?

- ☐ Straight ahead

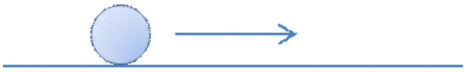

- ☐ Downhill

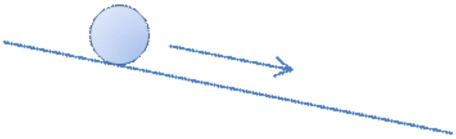

- ☐ Uphill

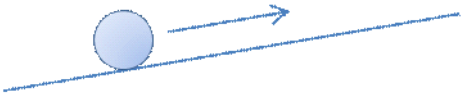

- ☐ Downhill

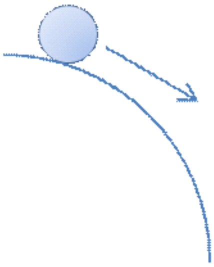

10. A motorcycle accelerates at a constant rate from 0 to 100 km/h. Thus the velocity increases linearly. Which of the following statements are true?

- ☐ The propulsive force needs to increase, independent of the air and friction resistance, to the same extent as the velocity.
- ☐ The propulsive force remains constant during acceleration, given that air and friction resistance do not change.
- ☐ The propulsive force is greatest at the beginning. It can then be reduced slowly as the motorcycle will increasingly utilize its own momentum for acceleration.
- ☐ When the air resistance increases with increasing velocity, the propulsive force also needs to increase accordingly.

11. The following three balls roll on a horizontal plane:

- Ball A rolls with velocity 1 m/s around a bend.
- Ball B starts with a velocity of 6 m/s, then its velocity continuously decreases.
- Ball C moves with an ever increasing velocity.

Which of the following statements are true?

☐ A horizontal force acts on ball A.

☐ A horizontal force acts on ball B.

☐ A horizontal force acts on ball C.

12. a) Two skaters with clearly different body weights stand opposite each other, each on their own skateboard, and are connected by a rope under tension. The lighter skater on the left pulls actively on the rope, while the heavier skater on the right just holds on to it. Which of the following statements are true?

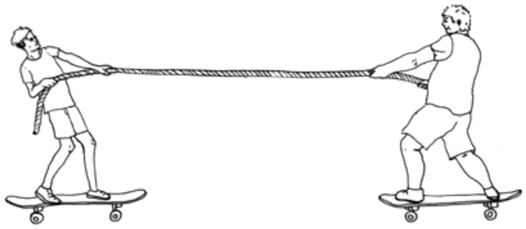

☐ They will meet at a point that is closer to the starting position of the lighter skater.

☐ Nothing happens, because the force of the pull results in an equally large counter-force, such that both forces cancel each other out.

☐ The lighter skater remains stationary and the heavier skater rolls towards him.

☐ Both move towards the middle at the same speed.

☐ They meet at a point that is closer to the starting position of the heavier skater.

b) Which of the following explanations for your answer(s) is correct? **Please tick only one answer.**

- ☐ As the left skater pulls on the right skater, and not the other way round, the right skater moves.
- ☐ Because the right skater holds on to the rope, the rope also transmits some pulling force to the left skater.
- ☐ The right skater has to hold on to the rope with a force that is equivalent to the force with which the left skater is pulling. Therefore, the same amount of force acts on both.
- ☐ The left skater is affected by his own force plus the force with which the right skater is holding on to the rope. Therefore the left skater moves faster than the right.
- ☐ The pulling force of the left skater is divided, through the rope, half to the left and half to the right skater.

List of correct answer alternatives of all items of the bMCU Test

Item	Answer alternatives correct
1. Water Glass	4
2. Book	2
3. Bus	1
4. Train	a3, b4
5. Hiker	2
6. Cart	1, 4
7. Object Motion	4, 5
8. Stone	3, 4
9. Inclined Plane	4
10. Motorcycle	2, 4
11. Balls	1, 2, 3
12. Skaters	a5, b3
