# Supplementary material for: The test of basic Mechanics Conceptual Understanding (bMCU): using Rasch analysis to develop and evaluate an efficient multiple choice test on Newton’s mechanics
Source: Int J STEM Educ. 2017 Sep 20;4(1):18. doi: 10.1186/s40594-017-0080-5 (PMC6310380; doi:10.1186/s40594-017-0080-5)
Supplement: Supplementary file 1 — English version and the original German version of the bMCU test. (ZIP 314 kb) [file 40594_2017_80_MOESM1_ESM.zip › bMCU_Test_German.pdf]

Sehr geehrte Testanwender,

Aufgabe 2 “Buch” sollte nicht verwendet werden, wenn Vergleiche über die Zeit beabsichtigt sind und der bMCU Test wiederholt eingesetzt wird, da diese Aufgabe für SchülerInnen mit und ohne Unterrichtserfahrungen nicht zuverlässig dasselbe Konstrukt misst. **Folglich sollte Aufgabe 2 bei Veränderungsmessungen nicht berücksichtigt werden**.

Wenn keine direkten Vergleiche über die Zeit beabsichtigt sind, kann der vollständige Test (d.h., alle 12 Aufgaben) verwendet werden.

Wichtiger Hinweis:

Bei den folgenden Fragen **können mehrere Antworten richtig sein**. Kreuzen Sie alle richtigen Antworten an.

Markieren Sie bitte ein deutliches Kreuz ins Kästchen: ☒

Machen Sie bitte die Kreuze mit einem Bleistift und drücken Sie fest auf, so dass man das Kreuz gut lesen kann. Wenn Sie korrigieren möchten, radieren Sie das Kreuz sauber aus.

Versuchen Sie, alle Aufgaben zu lösen. Halten Sie sich nicht zu lange bei einer einzelnen Aufgabe auf.

1. Ein volles Wasserglas steht stabil auf der Rückbank eines konstant geradeaus fahrenden Autos. Plötzlich tritt der Fahrer das Gaspedal durch und beschleunigt das Auto. Welche der folgenden Aussagen treffen zu?

   - ☐ Weil sich das Glas bezüglich der Rückbank im Auto nicht bewegt, bleibt die Wasseroberfläche unverändert.
   - ☐ Das Wasser wird mit dem Auto beschleunigt, so dass etwas Wasser in Fahrtrichtung über den Rand des Glases schwappt.
   - ☐ Aufgrund der Trägheit des Wassers verändert sich die Wasseroberfläche nicht.
   - ☐ Das Wasser behält zunächst seinen vorherigen Bewegungszustand bei, so dass etwas Wasser entgegen der Fahrtrichtung über den Rand des Glases schwappt.

2. Ein Buch liegt vor dir auf dem Tisch. Welche der folgenden Aussagen treffen zu?

   - ☐ Wie auf jeden anderen ruhenden Körper wirkt auf das Buch nur die Anziehungskraft der Erde.
   - ☐ Der Tisch stützt das Buch ab und wirkt deshalb mit einer nach oben gerichteten Kraft auf das Buch.
   - ☐ Da das Buch in Ruhe ist, kann hier überhaupt nicht mit dem Kraftbegriff argumentiert werden.
   - ☐ Auf das Buch wirkt nur die Stützkraft des Tisches, sonst würde es herunterfallen.

3. Ein Bus fährt mit konstanter Geschwindigkeit auf horizontaler Strasse geradeaus. Welche der folgenden Aussagen treffen zu?

   - ☐ Damit der Bus nicht langsamer wird, muss die Antriebskraft des Motors genau so gross sein wie der Luftwiderstand und die übrigen Reibungskräfte zusammen.
   - ☐ Damit die Geschwindigkeit konstant bleibt, muss die Antriebskraft grösser sein als der Luftwiderstand und die übrigen Reibungskräfte zusammen.
   - ☐ Damit die Geschwindigkeit nicht zunimmt, muss die Antriebskraft etwas geringer sein als der Luftwiderstand und die übrigen Reibungskräfte zusammen.
   - ☐ Die Antriebskraft ist nur zum Beschleunigen erforderlich, bei konstanter Geschwindigkeit hingegen nicht.

4. a) Ein Junge spielt im Gang des Wagons eines mit konstanter Geschwindigkeit geradeaus fahrenden Zuges mit seinem Ball. Welche Aussagen treffen zu?

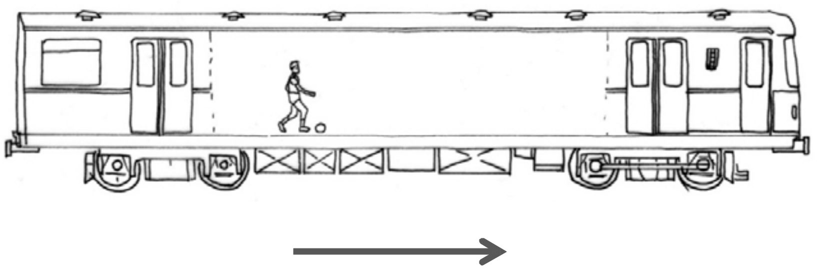

- ☐ Je nachdem, ob er den Ball aus der Mitte des Wagons in oder entgegen der Fahrtrichtung tritt, ist der Ball unterschiedlich schnell am Ende des Wagons.
- ☐ Wirft er den Ball in die Luft, muss er entweder entgegen oder in Fahrtrichtung laufen, um den Ball wieder aufzufangen.
- ☐ Der Ball verhält sich immer so, wie wenn der Zug stehen würde.
- ☐ Je nachdem, ob er den Ball in oder entgegen der Fahrtrichtung tritt, benötigt er zum Treten unterschiedlich viel Kraft.

b) Welche der folgenden Erklärungen liegt/liegen deiner/deinen Antwort(en) zugrunde?

- ☐ Sobald der Ball hochgeworfen wird, bleibt seine Bewegung hinter der des Wagons zurück. Der Grund dafür liegt in der Trägheit des Balls, die sich gegen die Bewegungsänderung stemmt.
- ☐ Wenn der Ball hochgeworfen wird, addiert sich die vertikale Bewegung vektoriell zu der horizontalen Bewegung in Fahrtrichtung. Deshalb bewegt sich der Ball vom Jungen in Fahrtrichtung weg.
- ☐ Um den Ball entgegen der Fahrtrichtung abzutreten, ist mehr Kraft erforderlich, als in Fahrtrichtung, weil man entgegen der Fahrtrichtung zusätzlich gegen die Bewegungsrichtung des Balls antreten muss.
- ☐ Aufgrund seiner Trägheit bewegt sich der Ball immer mit derselben horizontalen Geschwindigkeit wie der Wagon, sofern er nicht in oder entgegen der Fahrtrichtung getreten wird.
- ☐ Wird der Ball aus der Mitte nach vorne abgetreten, bewegt sich der vordere Teil des Wagons vom Ball weg. Wird der Ball nach hinten abgetreten, bewegt sich der hintere Teil des Wagons auf den Ball zu. Die Zeiten, bis der Ball jeweils das Ende des Wagons erreicht, sind daher verschieden.

5. Ein Wanderer hebt einen Stein auf und geht mit 1 m/s weiter. Nach kurzer Zeit lässt er den Stein im Gehen aus 1 Meter Höhe wieder fallen. Nach ½ Sekunde trifft der Stein wieder am Boden auf. Wo landet der Stein?

- ☐ Der Stein landet in etwa ½ Meter hinter dem Wanderer, weil der Wanderer in ½ Sekunde etwa ½ Meter zurücklegt.
- ☐ Der Stein landet in etwa neben den Füssen des Wanderers, da der Stein aufgrund seiner Trägheit seine horizontale Bewegung beibehält.
- ☐ Weil der Stein in einem nach hinten gerichteten Bogen zu Boden fällt, landet er in etwa 1 Meter hinter dem Wanderer.
- ☐ Da der Stein aufgrund seiner Trägheit seine horizontale Bewegung beibehält, landet er in etwa ½ Meter vor dem Wanderer.

6. Auf einem Modellwagen befindet sich eine Metallkugel, die auf einer Schiene ungehindert nach links und rechts rollen kann. An den Enden der Schiene sind Begrenzungen angebracht, die verhindern, dass die Kugel von der Schiene rollen kann.

In der Ausgangssituation ist die Kugel in der Mitte des Wagens.

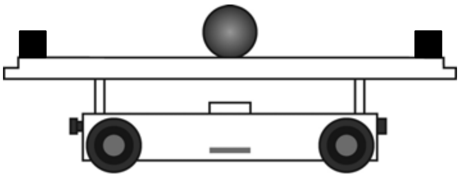

Was geschieht, wenn der Wagen aus dem Stillstand nach rechts angestossen wird?

- ☐ Die Kugel rollt auf der Schiene nach links entgegen der Fahrtrichtung.
- ☐ Die Kugel ändert ihre Position auf dem Wagen nicht.
- ☐ Die Kugel rollt auf der Schiene nach rechts in Fahrtrichtung.
- ☐ Die Kugel bleibt bezüglich der Tischoberfläche in etwa am selben Ort bis sie an der Begrenzung des Wagens anstösst.

7. Nachdem ein Körper angestossen wurde, gleitet er auf einer glatten Oberfläche reibungs- und luftwiderstandsfrei dahin. Welche Aussagen treffen zu?

- ☐ Der Schwung durch das Anstossen verbraucht sich mit der Zeit. Deshalb wird der Körper immer langsamer, bis er schliesslich zum Stillstand kommt.
- ☐ Da er sich bewegt, muss auf den Körper eine Kraft in Bewegungsrichtung wirken.
- ☐ Die Masse des Körpers wirkt der Bewegung entgegen. Je schwerer der Körper ist, desto schneller wird er zur Ruhe kommen.
- ☐ Der Körper gleitet mit konstanter Geschwindigkeit über die Oberfläche.
- ☐ Der Körper ändert seine Bewegung nicht, weil keine horizontale Kraft auf ihn wirkt.

8. Eine Person steht in einem ruhenden Boot und wirft mit Schwung einen grossen Stein ins Wasser hinter dem Boot. Welche Aussagen treffen zu?

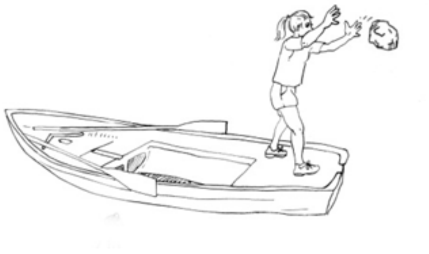

- ☐ Das Boot bewegt sich in Wurfrichtung des Steins.
- ☐ Der Stein verdrängt Wasser und dadurch schaukelt das Boot nur etwas hin und her.
- ☐ Lässt man einen aufgeblasenen Luftballon durch die Luft zischen, so passiert im Prinzip dasselbe.
- ☐ Das Boot bewegt sich entgegen der Wurfrichtung des Steins.

9. Die folgenden vier Abbildungen zeigen jeweils eine Kugel, die sich auf verschieden geneigten bzw. gekrümmten Bahnen reibungsfrei bewegt. Auf welchen dieser Abbildungen ändern sich im Laufe der dargestellten Bewegung die Kräfte, die auf die Kugel wirken?

- ☐ geradeaus

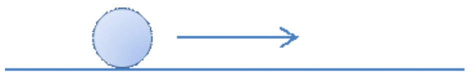

- ☐ bergab

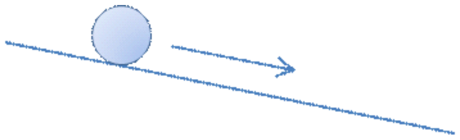

- ☐ bergauf

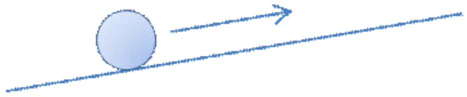

- ☐ bergab

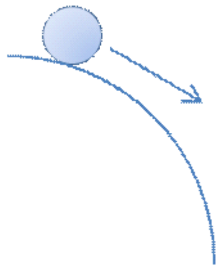

10. Ein Motorrad beschleunigt gleichmässig von 0 auf 100 km/h. Die Geschwindigkeit nimmt somit linear zu. Welche der folgenden Aussagen treffen zu?

- ☐ Die Antriebskraft muss unabhängig von Luft- und Reibungswiderständen in gleichem Mass wie die Geschwindigkeit zunehmen.
- ☐ Die Antriebskraft bleibt während des ganzen Beschleunigungsvorgangs konstant, vorausgesetzt, dass sich Luft- und Reibungswiderstände nicht ändern.
- ☐ Die Antriebskraft ist zu Beginn am grössten. Sie kann langsam reduziert werden, da das Motorrad zunehmend den Schwung zur Beschleunigung ausnutzen kann.
- ☐ Wenn der Luftwiderstand mit zunehmender Geschwindigkeit wächst, dann muss auch die Antriebskraft entsprechend zunehmen.

11. Die folgenden drei Kugeln bewegen sich auf einer waagrechten Ebene:

- Kugel A rollt mit der Geschwindigkeit 1 m/s um eine Kurve.
- Kugel B beginnt mit einer Geschwindigkeit von 6 m/s und wird dann immer langsamer.
- Kugel C bewegt sich immer schneller.

Welche der folgenden Aussagen treffen zu?

☐ Kugel A erfährt eine horizontale Kraft.

☐ Kugel B erfährt eine horizontale Kraft.

☐ Kugel C erfährt eine horizontale Kraft.

12. a) Zwei Skateboard-Fahrer mit deutlich unterschiedlichem Gewicht stehen sich je auf einem Skateboard gegenüber und sind mit einem gespannten Seil verbunden. Der linke und leichtere Skater zieht aktiv am Seil, der schwerere rechte Skater hält es nur fest. Was trifft zu?

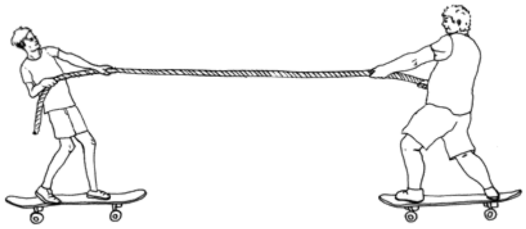

☐ Sie treffen sich in einem Punkt, der näher bei der Ausgangsposition des leichteren Skaters liegt.

☐ Es passiert nichts, da die Kraft des Zuges eine ebenso grosse Gegenkraft hervorruft und sich die beiden Kräfte somit aufheben.

☐ Der leichtere Skater bleibt stehen, der schwerere Skater rollt auf ihn zu.

☐ Beide bewegen sich gleich schnell zur Mitte hin.

☐ Sie treffen sich in einem Punkt, der näher bei der Ausgangsposition des schwereren Skaters liegt.

b) Welche der folgenden Erklärungen für deine Antwort(en) ist richtig? **Bitte kreuze nur eine Antwort an.**

- ☐ Da der linke Skater am rechten zieht und nicht umgekehrt, bewegt sich der rechte Skater.
- ☐ Weil der rechte Skater das Seil ebenfalls festhalten muss, übt das Seil auch einen geringeren Zug auf den linken Skater aus.
- ☐ Der rechte Skater muss das Seil genauso fest halten, wie der linke Skater am Seil zieht. Auf beide wirkt deshalb eine gleich grosse Kraft.
- ☐ Auf den linken Skater wirkt seine eigene Kraft plus diejenige, mit der der rechte das Seil hält. Deshalb muss sich der linke Skater schneller bewegen als der rechte.
- ☐ Die Zugkraft des linken Skaters wird über das Seil zur Hälfte auf den linken, zur Hälfte auf den rechten Skater aufgeteilt.

Liste der korrekten Antwortalternativen aller Aufgaben des bMCU Tests

Aufgabe	Korrekte Antwortalternativen
1. Wasserglas	4
2. Buch	2
3. Bus	1
4. Zug	a3, b4
5. Wanderer	2
6. Wagen	1, 4
7. Körper in Bewegung	4, 5
8. Stein	3, 4
9. Schiefe Ebene	4
10. Motorrad	2, 4
11. Kugeln	1, 2, 3
12. Skater	a5, b3
