# Supplementary material for: The test of basic Mechanics Conceptual Understanding (bMCU): using Rasch analysis to develop and evaluate an efficient multiple choice test on Newton’s mechanics
Source: Int J STEM Educ. 2017 Sep 20;4(1):18. doi: 10.1186/s40594-017-0080-5 (PMC6310380; doi:10.1186/s40594-017-0080-5)
Supplement: Supplementary file 2 — Additional Information on the Item Selection Process. (PDF 349 kb) [file 40594_2017_80_MOESM2_ESM.pdf]

Additional Information on the Item Selection Process

Item-fit statistics alone (i.e., statistics that examine the fit of individual items of a test), which are often used for item selection, are not well suited for this purpose (Edelsbrunner & Dablander, 2015; Karabatsos, 2000; Smith & Plackner, 2009). They might not detect violations of the assumptions of one-dimensionality (Smith, Schumacker, & Bush, 1998) and subgroup homogeneity (Smith & Suh, 2003). Moreover, evaluating single items' deviations from the Rasch model is considered unreasonable without conducting a global test of whether the Rasch model holds (Christensen & Kreiner, 2013). To guarantee valid item selection, we therefore applied different test statistics, starting with global and continuing with local (i.e., item-specific) analyses.

We also fitted Rasch mixture models (Rost & von Davier, 1995), which search for latent classes within a sample indicated by maximally different item parameter estimations. These latent classes represent students who answer items systematically differently, in other words, for whom the test does not measure the same underlying construct. When models with more than one class were shown to better fit the data than the regular (one-class) Rasch model did, we inspected the item difficulties estimated for the latent classes to determine which items particularly differed between the detected latent classes. We used the package Psychomix (Frick, Strobl, Leisch, & Zeileis, 2012) to test Rasch mixture models.

Christensen, K. B., & Kreiner, S. (2013) Item fit statistics. In K. B. Christensen, S. Kreiner, & M. Mesbah (Eds.), *Rasch models in health* (pp. 83–103). Hoboken, NJ: John Wiley & Sons.

Edelsbrunner, P. A., & Dablander, F. (2015). Inconsistent Rasch modeling practices in research on scientific reasoning. Manuscript in preparation.

Frick, H., Strobl, C., Leisch, F., & Zeileis, A. (2012). Flexible Rasch mixture models with package psychomix. *Journal of Statistical Software, 48*(7), 1–25.

Karabatsos, G. (2000). A critique of Rasch residual fit statistics. *Journal of Applied Measurement, 1*, 152–176.

Rost, J., & von Davier, M. (1995). Mixture distribution Rasch models. In G. H. Fischer & I. W. Molenaar (Eds.), *Rasch models* (pp. 257–268). Springer New York. Retrieved from http://link.springer.com/chapter/10.1007/978-1-4612-4230-7_14

Smith, R. M., & Plackner, C. (2009). The family approach to assessing fit in Rasch measurement. *Journal of Applied Measurement, 10*, 424–437.

Smith, R. M., Schumacker, R. E., & Bush, M. J. (1998). Using item mean squares to evaluate fit to the Rasch model. *Journal of Outcome Measurement, 2*, 66–78.

Smith, R. M., & Suh, K. K. (2003). Rasch fit statistics as a test of the invariance of item parameter estimates. *Journal of Applied Measurement, 4*, 153–163.
