# Supplementary material for: The test of basic Mechanics Conceptual Understanding (bMCU): using Rasch analysis to develop and evaluate an efficient multiple choice test on Newton’s mechanics
Source: Int J STEM Educ. 2017 Sep 20;4(1):18. doi: 10.1186/s40594-017-0080-5 (PMC6310380; doi:10.1186/s40594-017-0080-5)
Supplement: Supplementary file 3 — Additional Information on the Test Evaluation Process. (PDF 353 kb) [file 40594_2017_80_MOESM3_ESM.pdf]

Additional Information on the Test Evaluation Process

To further examine subgroup homogeneity, Rasch mixture models (Rost & von Davier, 1995) with two and three classes were compared to the solution with only one class (i.e., the Rasch model). A better fit of a model with more than one class would indicate that there are specific classes of students who exhibit systematically different answering patterns. We used the package Psychomix (Frick, Strobl, Leisch, & Zeileis, 2012) to test Rasch mixture models.

The comparison of Rasch mixture models with two and three classes to the solution with only one class (the Rasch model) additionally underpinned subgroup homogeneity with both the Bayesian information criterion (BIC) and the integrated classification likelihood (ICL) favoring the one-class solution.

Applying the packages eRm (Mair, Hatzinger, & Maier, 2013) and ltm (Rizopoulos, 2006), the Rasch model was compared with the two-parameter Birnbaum model, which includes a second parameter allowing the items to differ in discrimination. The less restrictive Birnbaum model should not fit the data better than the parsimonious Rasch model. Otherwise, the characteristics of a Rasch-scaled test might not hold true for the new instrument.

The less restrictive Birnbaum model did not fit the data better than the parsimonious Rasch model. This result indicated that we did not have to include an item discrimination parameter in the model (as is the case in the Birnbaum model) that enables the items to contribute to a different degree to the construct measured. We could thus adhere to the more restrictive Rasch model, which specifies that every item contributes to the same extent to the construct measured.

Frick, H., Strobl, C., Leisch, F., & Zeileis, A. (2012). Flexible Rasch mixture models with package psychomix. *Journal of Statistical Software, 48*(7), 1–25.

Mair, P., Hatzinger, R., & Maier, M. J. (2013). *eRm: Extended Rasch Modeling. R package version 0.15-3.* Retrieved from http://CRAN.R-project.org/package=eRm

Rizopoulos, D. (2006). ltm: An R package for latent variable modeling and item response theory analyses. *Journal of Statistical Software, 17*(5), 1–25.

Rost, J., & von Davier, M. (1995). Mixture distribution Rasch models. In G. H. Fischer & I. W. Molenaar (Eds.), *Rasch models* (pp. 257–268). Springer New York. Retrieved from http://link.springer.com/chapter/10.1007/978-1-4612-4230-7_14
