# Supplementary figures and images for: The test of basic Mechanics Conceptual Understanding (bMCU): using Rasch analysis to develop and evaluate an efficient multiple choice test on Newton’s mechanics
Source: Int J STEM Educ. 2017 Sep 20;4(1):18. doi: 10.1186/s40594-017-0080-5 (PMC6310380; doi:10.1186/s40594-017-0080-5)

Figure S1. Scree plot showing the Eigenvalues for different factor solutions on bMCU Test data.

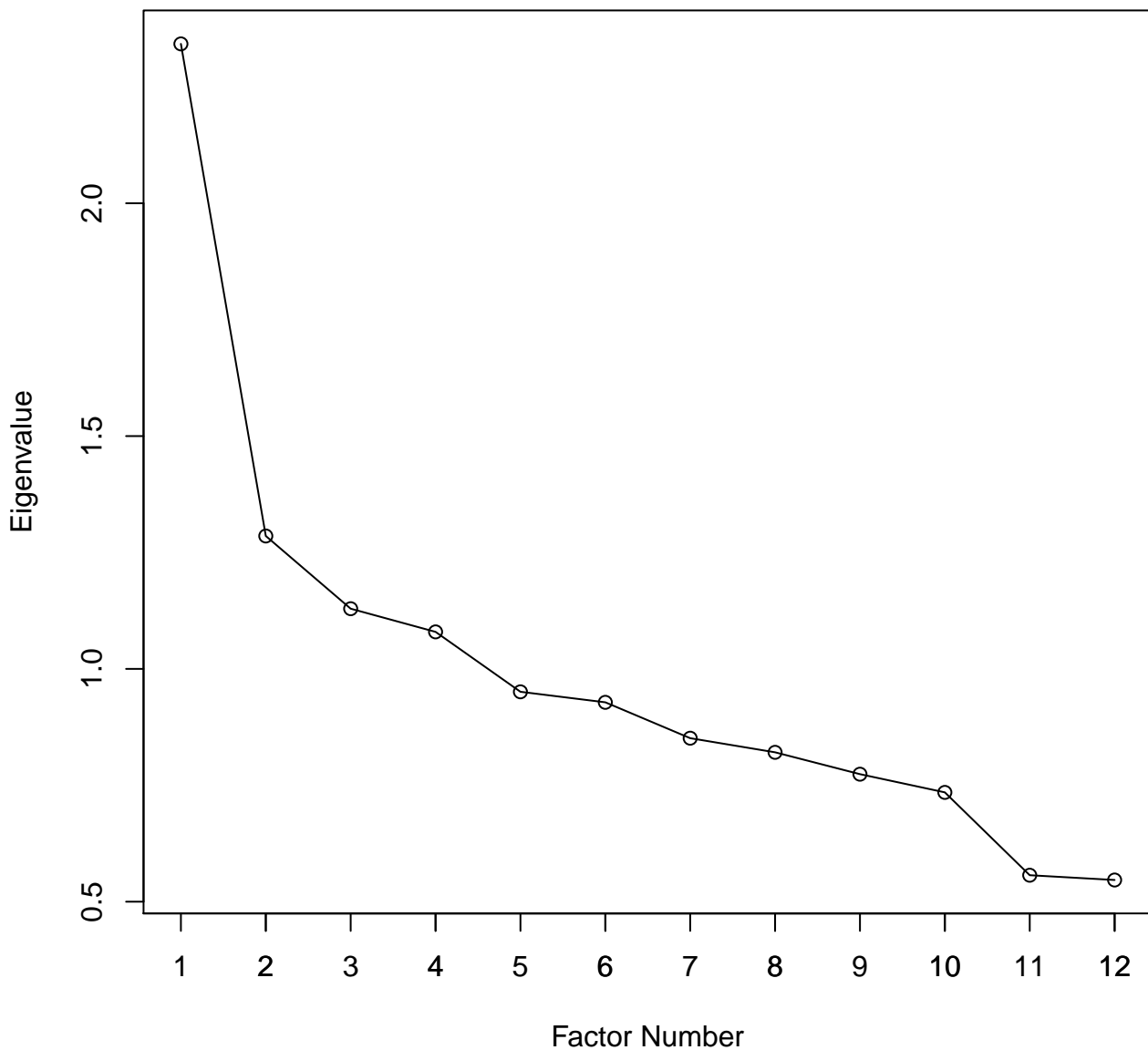

Supplement: Supplementary file 4 — Scree plot showing the eigenvalues for different factor solutions on bMCU test data. (PDF 38 kb) [file 40594_2017_80_MOESM4_ESM.pdf]

Figure S3. Wright Map on the 11-item version of the bMCU Test and the  $N = 249$  students.

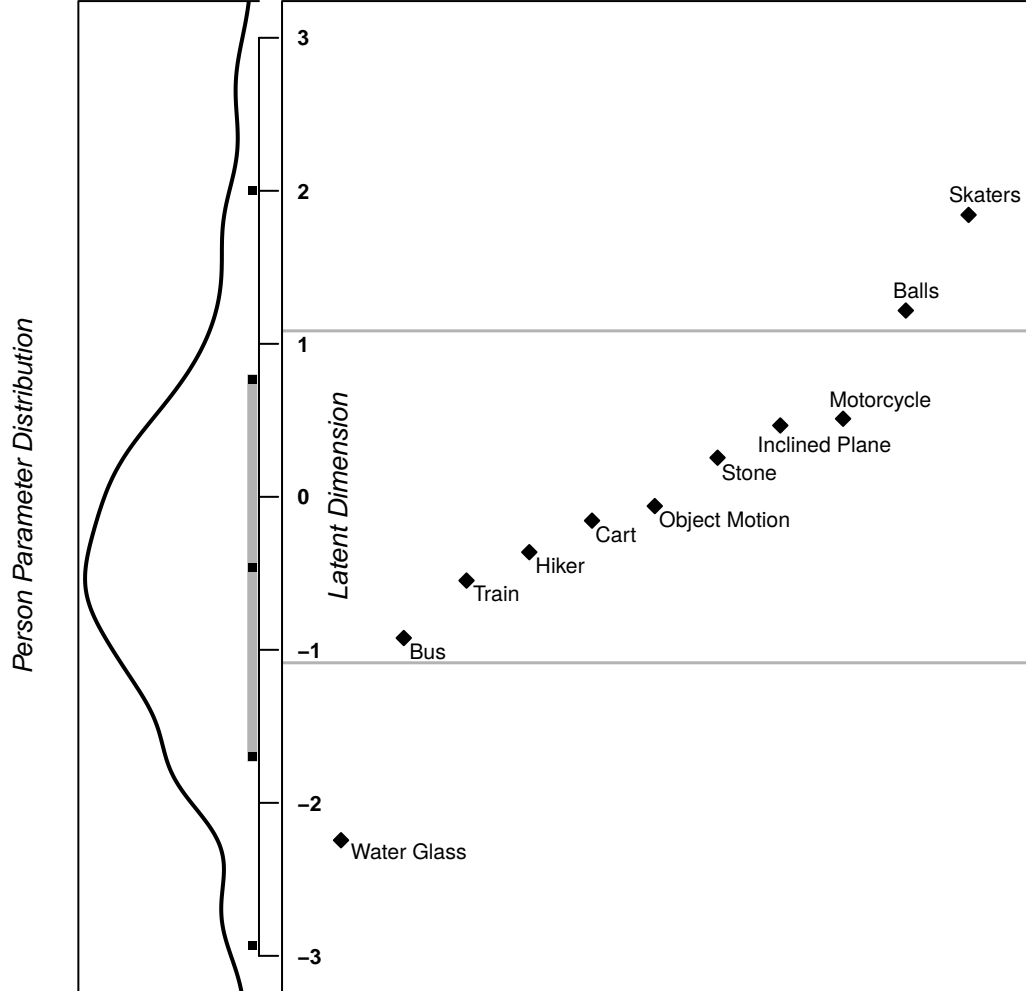

Supplement: Supplementary file 8 — Wright Map on the 11-item version of the bMCU test and the N = 249 students. (PDF 80 kb) [file 40594_2017_80_MOESM8_ESM.pdf]
