# Supplementary material for: The test of basic Mechanics Conceptual Understanding (bMCU): using Rasch analysis to develop and evaluate an efficient multiple choice test on Newton’s mechanics
Source: Int J STEM Educ. 2017 Sep 20;4(1):18. doi: 10.1186/s40594-017-0080-5 (PMC6310380; doi:10.1186/s40594-017-0080-5)
Supplement: Supplementary file 5 — Factor loadings of the 12 items of the bMCU test given a one-factor, a two-factor, and a three-factor solution based on the sample of N = 249 students. (PDF 55 kb) [file 40594_2017_80_MOESM5_ESM.pdf]

Table S1. Factor loadings of the 12 items of the bMCU Test given a one-factor, a two-factor, and a three-factor solution based on the sample of $N = 249$ students

| Item | One-factor solution | Two-factor solution | | Three-factor solution | | |
|---|---|---|---|---|---|---|
| | | 1 | 2 | 1 | 2 | 3 |
| 1. Water Glass | 0.301 | 0.104 | 0.274 | 0.080 | 0.061 | 0.476 |
| 2. Book | 0.253 | 0.189 | 0.155 | -0.038 | 0.172 | 0.239 |
| 3. Bus | 0.336 | 0.119 | 0.347 | 0.087 | 0.121 | 0.471 |
| 4. Train | 0.340 | 0.045 | 0.483 | 0.997 | 0.029 | 0.006 |
| 5. Hiker | 0.425 | 0.084 | 0.612 | 0.322 | 0.143 | 0.324 |
| 6. Cart | 0.281 | 0.292 | 0.078 | -0.007 | 0.253 | 0.165 |
| 7. Object Motion | 0.380 | 0.272 | 0.264 | 0.149 | 0.281 | 0.170 |
| 8. Stone | 0.294 | 0.389 | 0.027 | -0.027 | 0.346 | 0.114 |
| 9. Inclined Plane | 0.324 | 0.276 | 0.168 | 0.153 | 0.259 | 0.096 |
| 10. Motorcycle | 0.468 | 0.676 | 0.073 | 0.052 | 0.806 | -0.055 |
| 11. Balls | 0.441 | 0.370 | 0.213 | 0.172 | 0.326 | 0.177 |
| 12. Skaters | 0.300 | 0.189 | 0.239 | 0.150 | 0.208 | 0.134 |

Notes: The results are based on maximum likelihood factor analyses with varimax rotation.
