# Supplementary material for: The test of basic Mechanics Conceptual Understanding (bMCU): using Rasch analysis to develop and evaluate an efficient multiple choice test on Newton’s mechanics
Source: Int J STEM Educ. 2017 Sep 20;4(1):18. doi: 10.1186/s40594-017-0080-5 (PMC6310380; doi:10.1186/s40594-017-0080-5)
Supplement: Supplementary file 6 — Fit of the Rasch model for the 11-item version of the bMCU test. (PDF 318 kb) [file 40594_2017_80_MOESM6_ESM.pdf]

Fit of the Rasch Model for the 11-Item Version of the bMCU Test

In the following section, the findings of the evaluation of the 11-item version of the bMCU Test are briefly summarized. We used the same sample (N = 141 students) and the same procedure that was used for the evaluation of the full 12-item version. It becomes clear that Rasch model conformity can also be ascertained for the 11-item version of the bMCU Test.

Concerning general model fit, Pearson's χ^2-goodness-of-fit (bootstrap) test suggested conformity of the data to the Rasch model (p = .27). Andersen's conditional likelihood ratio tests and all of the nonparametric T10-statistics (including the examination of the effects of re-testing) indicated subgroup homogeneity with all ps ≥ .05 (see Table S2). The comparison of Rasch mixture models with two and three classes to the solution with only one class (the Rasch model) additionally underpinned subgroup homogeneity with both the Bayesian information criterion (BIC) and the integrated classification likelihood (ICL) favoring the one-class solution.

The nonparametric version of the Martin-Löf test confirmed that all of the item-subsets tested against one another measured the same underlying dimension. Thus, the exact p-value was estimated at p = .68 when comparing the first half of the items to the second half. Using the median of the item-specific solution rates as a split criterion, an exact p-value of p = .14 resulted. The exact p-value was p = .70 when comparing odd items to even items. In line with these results, a maximum likelihood factor analysis with varimax rotation could substantiate the fit of the data of the 11-item version to a one-factor solution (χ^2 = 52.46, df = 44, p = .18). Figure S2 provides the corresponding Scree plot and Table S3 lists the factor loadings of the 11 items of the 11-item version of the bMCU Test given a one-factor, a two-factor, and a three-factor solution. Moreover, the global nonparametric T11-statistic (p = .19) and Yen's Q3 (maximum value of Q3 < .16) suggested model fit when testing for local stochastic

independence. Finally, the less restrictive Birnbaum model did not fit the data better than the parsimonious Rasch model.

The results concerning the item parameter estimation are presented in Table S4. To base the item parameter estimation on a sufficiently large dataset, we applied the sample used to examine the effects of re-testing ($N = 249$) because no influence of repeated vs. one-time testing on the estimation of the item parameters could be observed (see Table S2). The item parameters estimated for the 11-item version closely resemble the parameters estimated for the full version.

Figure S3 provides a Wright Map or person-item map on the data of the 11-item version. This Wright Map closely resembles the Map of the 12-item version. Because there are no considerable differences, they can be interpreted in the same way.

In conclusion, the two versions of the bMCU Test are highly comparable. Both versions satisfy the Rasch model. Their correlation ranges between $r = .93$ and $r = .98$.
