# Supplementary material for: The test of basic Mechanics Conceptual Understanding (bMCU): using Rasch analysis to develop and evaluate an efficient multiple choice test on Newton’s mechanics
Source: Int J STEM Educ. 2017 Sep 20;4(1):18. doi: 10.1186/s40594-017-0080-5 (PMC6310380; doi:10.1186/s40594-017-0080-5)

Figure S2. Scree plot showing the Eigenvalues for different factor solutions on the 11-item version of the bMCU Test.

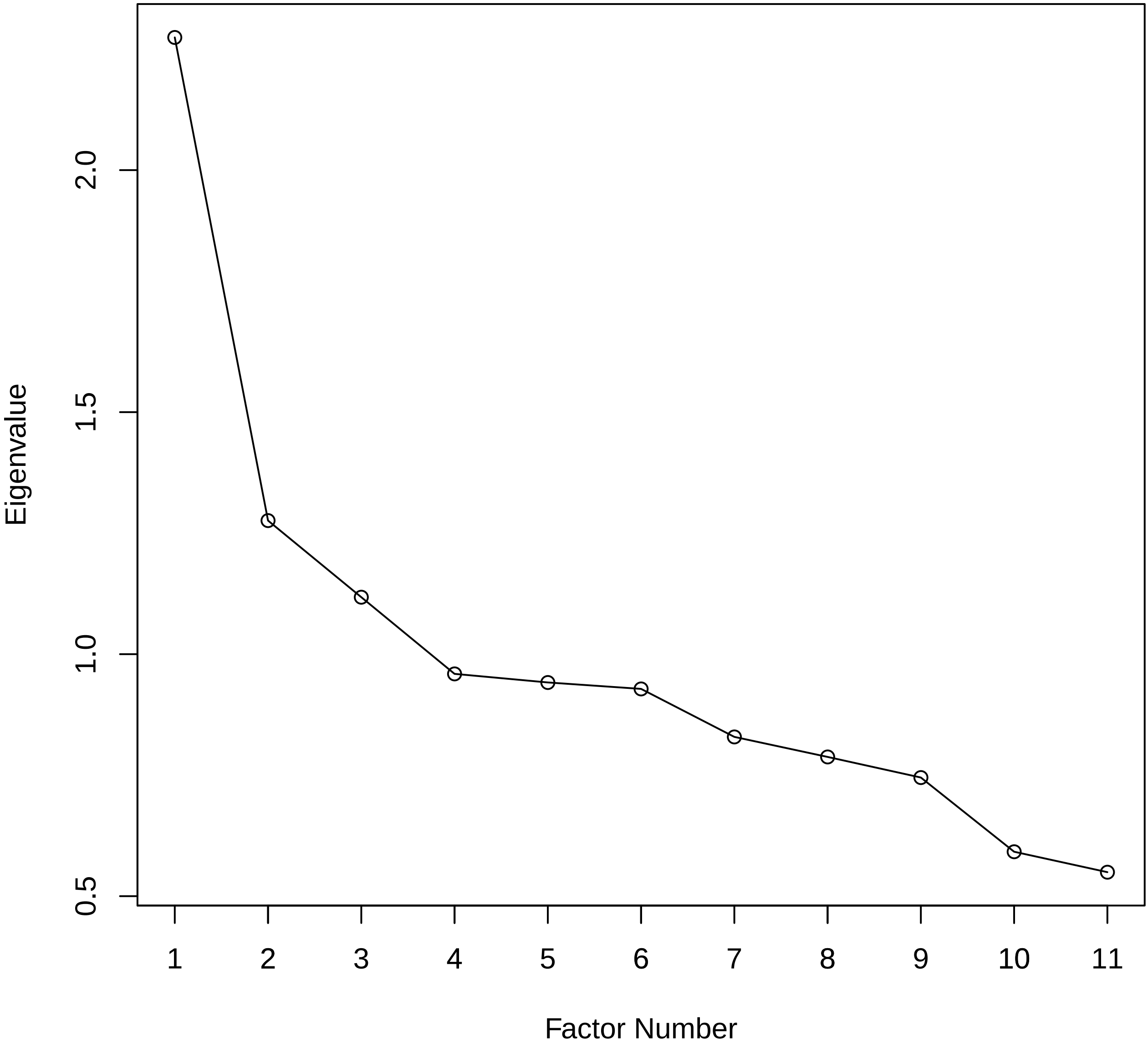

Supplement: Supplementary file 7 — Scree plot showing the eigenvalues for different factor solutions on the 11-item version of the bMCU test. (PDF 39 kb) [file 40594_2017_80_MOESM7_ESM.pdf]
