# Supplementary material for: The test of basic Mechanics Conceptual Understanding (bMCU): using Rasch analysis to develop and evaluate an efficient multiple choice test on Newton’s mechanics
Source: Int J STEM Educ. 2017 Sep 20;4(1):18. doi: 10.1186/s40594-017-0080-5 (PMC6310380; doi:10.1186/s40594-017-0080-5)
Supplement: Supplementary file 9 — Results of Andersen’s conditional likelihood ratio tests and the nonparametric T10-statistics with different split variables for the 11 items of the 11-item version without item 2 “Book”. (PDF 231 kb) [file 40594_2017_80_MOESM9_ESM.pdf]

Table S2. Results of Andersen's conditional likelihood ratio tests and the nonparametric T10-statistics with different split variables for the 11 items of the 11-item version without item 2 "Book"

	Subgroup size			
Split variable	n_1	n_2	p-value Andersen	p-value T10
Gender	69	72	.07	.05
Type of instruction	83	58	.17	.20
bMCU measure median	70	71	.33	.61
Age median	48	93	.72	.62
Intelligence (set II) median	61	64	.81	.65
Re-testing	108	141	.22	.24

Notes: Andersen's conditional likelihood ratio test and the nonparametric T10-statistic gauge the homogeneity in the item difficulty parameter estimates between subgroups. The subgroups are determined by the six split variables. All nonparametric statistics are based on n = 5000 sampled matrices. A non-significant p-value indicates no significant differences between subgroups in the item-difficulty parameter estimation. N = 16 students of the total sample of N = 141 students were missing when intelligence was assessed. In the last row, the results of the examination of the effects of re-testing are presented. DIF is examined in a sample of N = 249 students when one-time (n_1 = 108) vs. repeated testing (n_2 = 141) are compared.
