# Supplementary material for: The test of basic Mechanics Conceptual Understanding (bMCU): using Rasch analysis to develop and evaluate an efficient multiple choice test on Newton’s mechanics
Source: Int J STEM Educ. 2017 Sep 20;4(1):18. doi: 10.1186/s40594-017-0080-5 (PMC6310380; doi:10.1186/s40594-017-0080-5)
Supplement: Supplementary file 10 — Factor loadings of the 11 items of the 11-item version of the bMCU test given a one-factor, a two-factor, and a three-factor solution based on the sample of N = 249 students. (PDF 55 kb) [file 40594_2017_80_MOESM10_ESM.pdf]

Table S3. Factor loadings of the 11 items of the 11-item version of the bMCU Test given a one-factor, a two-factor, and a three-factor solution based on the sample of $N = 249$ students

Item	One-factor solution	Two-factor solution		Three-factor solution		
		1	2	1	2	3
1. Water Glass	0.298	0.070	0.301	0.067	0.080	0.505
3. Bus	0.335	0.108	0.357	0.130	0.097	0.486
4. Train	0.362	0.066	0.495	-0.004	0.902	0.019
5. Hiker	0.413	0.100	0.566	0.123	0.359	0.268
6. Cart	0.285	0.267	0.094	0.252	0.001	0.181
7. Object Motion	0.386	0.274	0.266	0.271	0.176	0.161
8. Stone	0.272	0.356	0.022	0.338	-0.018	0.088
9. Inclined Plane	0.319	0.262	0.174	0.243	0.175	0.077
10. Motorcycle	0.468	0.754	0.044	0.828	0.088	-0.072
11. Balls	0.454	0.344	0.239	0.313	0.195	0.186
12. Skaters	0.293	0.193	0.233	0.197	0.176	0.106

Notes: The results are based on maximum likelihood factor analyses with varimax rotation.
