# Supplementary material for: The test of basic Mechanics Conceptual Understanding (bMCU): using Rasch analysis to develop and evaluate an efficient multiple choice test on Newton’s mechanics
Source: Int J STEM Educ. 2017 Sep 20;4(1):18. doi: 10.1186/s40594-017-0080-5 (PMC6310380; doi:10.1186/s40594-017-0080-5)
Supplement: Supplementary file 11 — Item difficulty D i, standard error of D i, 95% confidence interval of D i, and outfit mean-square (MNSQ) for the 11 items of the 11-item version without item 2 “Book”. (PDF 168 kb) [file 40594_2017_80_MOESM11_ESM.pdf]

Table S4. Item difficulty D_i, standard error of D_i, 95% confidence interval of D_i, and outfit mean-square (MNSQ) for the 11 items of the 11-item version without item 2 "Book"

Item	Item difficulty D_i	Standard error	95%-CI		Outfit MNSQ
			LL	*UL*	
1. Water Glass	-2.24	0.17	-2.58	-1.91	0.90
3. Bus	-0.92	0.14	-1.19	-0.66	0.98
4. Train	-0.55	0.13	-0.81	-0.29	0.99
5. Hiker	-0.36	0.13	-0.62	-0.10	0.87
6. Cart	-0.16	0.13	-0.42	0.11	1.12
7. Object Motion	-0.06	0.13	-0.32	0.20	0.96
8. Stone	0.26	0.14	-0.01	0.53	1.16
9. Inclined Plane	0.47	0.14	0.19	0.74	1.03
10. Motorcycle	0.51	0.14	0.23	0.79	0.91
11. Balls	1.22	0.16	0.90	1.54	0.78
12. Skaters	1.84	0.19	1.46	2.22	0.84

Notes: CI = confidence interval; *LL* = lower limit, *UL* = upper limit. Item difficulty parameter D_i and its standard error estimated according to the Rasch model. Higher positive values indicate higher difficulty. Confidence intervals provide an idea of the precision of the difficulty-parameter estimation. Outfit MNSQ is a fit statistic comparing expected (based on the model) with observed data patterns that is sensitive to outliers. Values of approximately 1.00 (~ 0.50-1.50) indicate reasonable fit. The values obtained for the 11 items all fall within this range, indicating reasonable fit of the item data to the Rasch model.
