# Supplementary material for: The test of basic Mechanics Conceptual Understanding (bMCU): using Rasch analysis to develop and evaluate an efficient multiple choice test on Newton’s mechanics
Source: Int J STEM Educ. 2017 Sep 20;4(1):18. doi: 10.1186/s40594-017-0080-5 (PMC6310380; doi:10.1186/s40594-017-0080-5)

Figure S4. Item information curves for the 11 items of the 11-item version of the bMCU Test as a function of the person parameters of the *N* = 249 students.

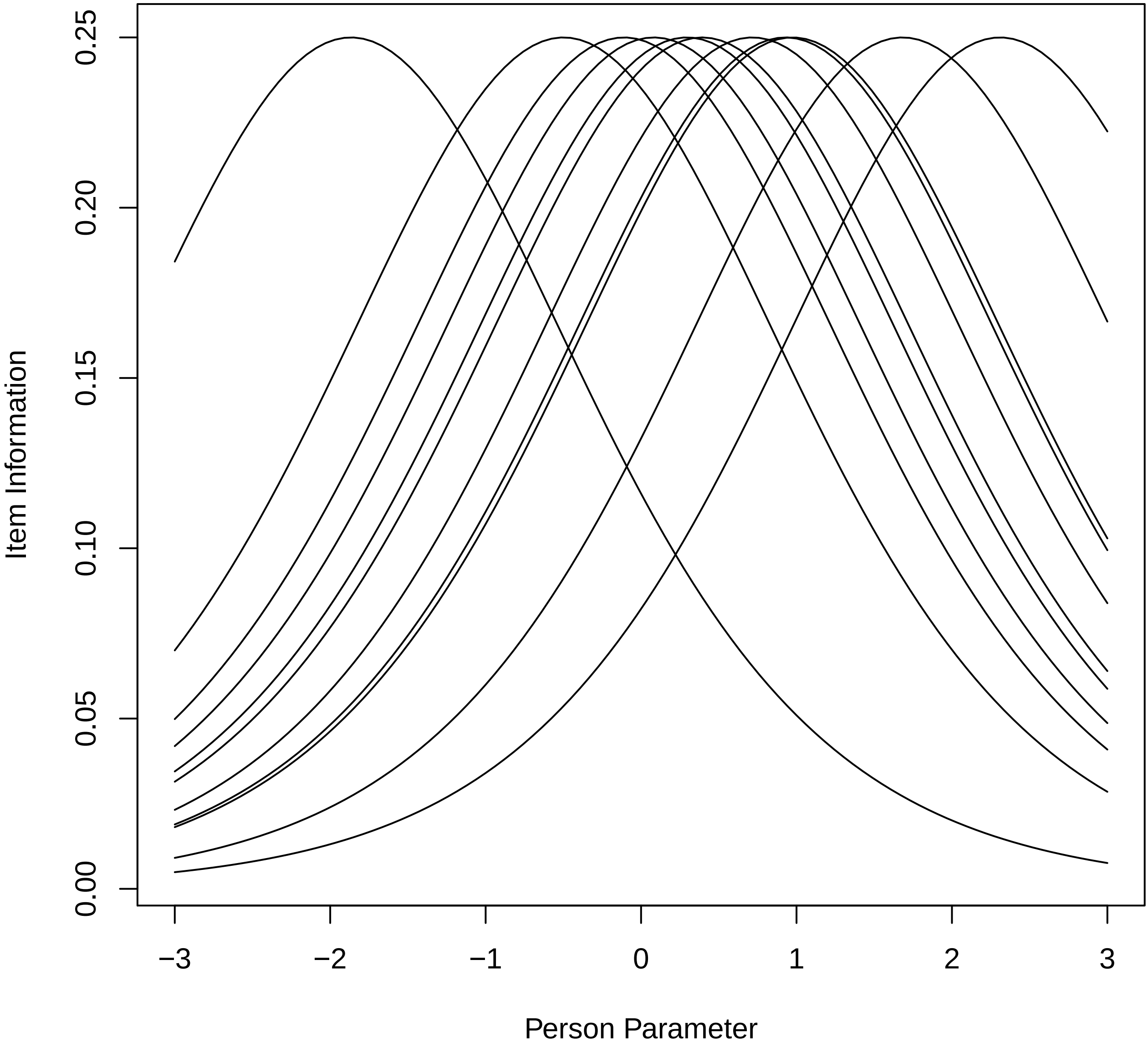

Supplement: Supplementary file 12 — Item information curves for the 11 items of the 11-item version of the bMCU test as a function of the person parameters of the N = 249 students. (PDF 60 kb) [file 40594_2017_80_MOESM12_ESM.pdf]
